# Targeted inhibition of the COP9 signalosome for treatment of cancer

**DOI:** 10.1038/ncomms13166

**Published:** 2016-10-24

**Authors:** Anita Schlierf, Eva Altmann, Jean Quancard, Anne B. Jefferson, René Assenberg, Martin Renatus, Matthew Jones, Ulrich Hassiepen, Michael Schaefer, Michael Kiffe, Andreas Weiss, Christian Wiesmann, Richard Sedrani, Jörg Eder, Bruno Martoglio

**Affiliations:** 1 Novartis Institutes for Biomedical Research, Novartis Pharma AG, 4002 Basel, Switzerland

## Abstract

The COP9 signalosome (CSN) is a central component of the activation and remodelling cycle of cullin-RING E3 ubiquitin ligases (CRLs), the largest enzyme family of the ubiquitin–proteasome system in humans. CRLs are implicated in the regulation of numerous cellular processes, including cell cycle progression and apoptosis, and aberrant CRL activity is frequently associated with cancer. Remodelling of CRLs is initiated by CSN-catalysed cleavage of the ubiquitin-like activator NEDD8 from CRLs. Here we describe CSN5i-3, a potent, selective and orally available inhibitor of CSN5, the proteolytic subunit of CSN. The compound traps CRLs in the neddylated state, which leads to inactivation of a subset of CRLs by inducing degradation of their substrate recognition module. CSN5i-3 differentially affects the viability of tumour cell lines and suppresses growth of a human xenograft in mice. Our results provide insights into how CSN regulates CRLs and suggest that CSN5 inhibition has potential for anti-tumour therapy.

## Abstract (web summary)

Dysregulation of protein degradation by the ubiquitin-proteasome system is a feature commonly associated with cancer. Here, the authors develop an orally available small molecule that inhibits CSN5, the proteolytic subunit of the COP9 signalosome, and blocks tumour growth in a xenograft model.

The ubiquitin–proteasome system (UPS) promotes the selective turnover of the majority of regulatory proteins within cells^1^. By a cascade of three enzymatic reactions, individual proteins are tagged with specific types of ubiquitin chains, which serve to direct proteins for degradation by the proteasome^2^,^3^. For the assembly of ubiquitin chains on target proteins, ubiquitin is first activated by an E1 ubiquitin-activating enzyme, transferred to an E2 ubiquitin-conjugating enzyme and eventually attached to the target protein by an E3 ubiquitin ligase^4^,^5^,^6^. Many proteins that are regulated by the UPS are central to tumorigenesis and tumour progression, and dysregulation of the UPS is frequently associated with cancer^7^,^8^. The therapeutic value of drugs targeting the UPS for the treatment of cancer is proven by the success of bortezomib and carfilzomib, which target the system at its very centre by inhibiting the proteolytic activity of the proteasome^9^,^10^. Bortezomib is approved for the treatment of multiple myeloma and mantle cell lymphoma, and carfilzomib for treatment of relapsed or refractory multiple myeloma. However, their use is limited by their narrow therapeutic window due to the broad biological effects seen on general proteasome inhibition^11^,^12^. One approach to more specifically inhibit the UPS is to target regulatory proteins that modulate UPS activity or interfere with substrate recognition and recruitment^13^,^14^.

E3 ubiquitin ligases define the substrate specificity of the UPS and comprise the largest enzyme family of the system with more than 600 putative members encoded by the human genome^15^. Within this family, cullin-RING E3 ubiquitin ligases (CRLs) are the largest subfamily, responsible for ∼20% of total cellular protein turnover^4^,^16^,^17^. CRLs are modular assemblies built around a central cullin scaffold, which associates with an adaptor protein, a substrate receptor module (SRM) and a RING protein that recruits the E2 enzyme^18^ (Fig. 1a). In mammals, about 200 SRMs are available to associate with one of eight cullins to form a CRL specific for a single or small group of substrate proteins^19^,^20^. Many different CRLs are simultaneously active within a cell at any time and the modular organization allows a dynamic assembly, disassembly and remodelling of CRLs corresponding to the cell's temporal requirements^21^. Critical steps in the cyclic regulation of CRLs are the activation of CRLs induced by the covalent attachment of the ubiquitin-like activator NEDD8 to the cullin moiety and its proteolytic removal leading to deactivation and disassembly^22^,^23^,^24^ (Fig. 1a). CRL neddylation is catalysed by the NEDD8-activating enzyme^25^ (NAE1) and induces a conformational rearrangement that enables the transfer of ubiquitin from the E2 enzyme to the ubiquitin-receiving substrate, which is recruited by the SRM of the respective CRL. The reverse reaction, deneddylation, is catalysed by the COP9 signalosome^26^,^27^,^28^ (CSN) and allows subsequent binding of factors that mediate the disassembly and remodelling of CRL complexes^24^.

The human CSN consists of eight protein subunits (CSN1–8), of which CSN5 contains a JAMM Zn^2+^-metalloprotease motif that provides the catalytic centre to the complex^27^,^29^,^30^. CSN5 exhibits proper deneddylating activity only in the context of the holocomplex and only the fully assembled CSN is competent to specifically remove NEDD8 from CRLs^30^. Multiple studies using RNA interference-induced CSN5 knockdown identified the protease as a positive regulator of oncogenes and negative regulator of tumour suppressors^31^,^32^,^33^,^34^. Its role in tumorigenesis is most likely to be indirect via the dysregulation of CRLs and, as a consequence, the disturbed balance of oncogenes and tumour suppressors controlled by CRLs. Furthermore, elevated expression of CSN5 and other CSN subunits is found in a number of human tumours, often correlating with poor prognosis^35^,^36^. These findings strongly qualify CSN5 as a potential drug target for anti-cancer therapy^37^. In the present study we describe the first selective small molecule inhibitor of CSN5. The compound potently inhibits the deneddylating activity of the CSN. We show how pharmacologic inhibition of CSN modulates CRLs *in vitro* and demonstrate the potential therapeutic value of an orally available CSN5 inhibitor in a human xenograft model.

## Results

### Discovery of selective CSN5 inhibitors

**Figure 1:**
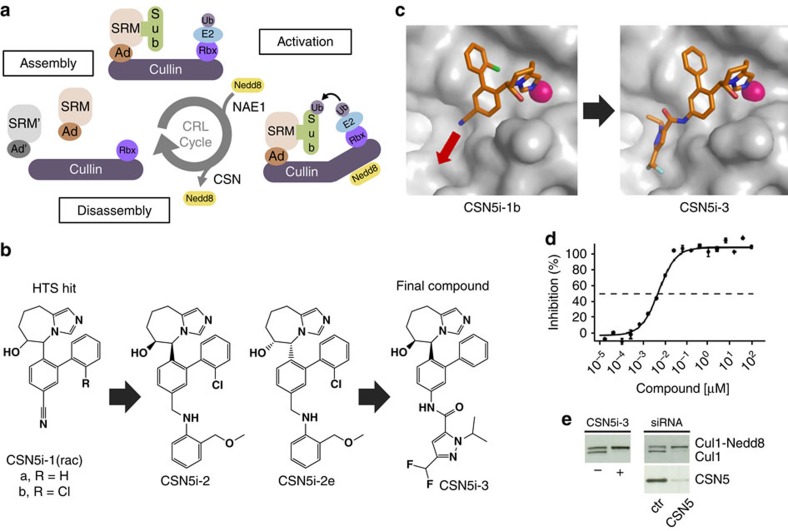
CSN5i-3 is a potent inhibitor of CSN5-catalysed cullin deneddylation. (**a**) Schematic illustration of the CRL cycle and the role of cullin neddylation and deneddylation. Ad, adaptor protein; SRM, interchangeable substrate recognition module; Sub, substrate. (**b**) Chemical structures of CSN5 inhibitors illustrating the optimization of the high throughput screening (HTS) hit CSN5i-1a to the cell active intermediate CSN5i-2, its *R,R*-enantiomer CSN5i-2e and to the final compound CSN5i-3. (**c**) Co-crystal structures of CSN5 with CSN5i-1b and CSN5i-3. The structures revealed the direction for compound extension into the substrate binding cleft of CSN5 (red arrow). (**d**) Dose–response curve for inhibition of CSN activity by CSN5i-3 as measured in a biochemical enzyme assay (*n*=2, ±s.d.). (**e**) Immunoblotting for Cul1 after treatment of HCT116 cells with 1 μM CSN5i-3 and for Cul1 and CSN5 after treatment with CSN5 siRNA.

CSN5 is a metalloprotease that exhibits deneddylating activity only in the context of the fully assembled CSN and only against NEDD8-conjugated CRLs^30^,^38^. Unlike the majority of proteases, it is not active as an individual subunit or as an isolated catalytic domain. Moreover, CSN does not cleave NEDD8-based peptide substrates, different to other deubiquitinating (DUB) enzymes, which typically hydrolyse ubiquitin-conjugated peptides^39^. For the identification of CSN inhibitors, we therefore conducted a high-throughput screening campaign using a biochemical assay with recombinant CSN and a fluorescence-labelled and NEDD8-modified CRL substrate^30^,^40^ (PT22-NEDD8-SCF^Skp2^; Supplementary Table 1). Among the hits, which included weakly active metal binders like hydroxamates, we selected the tetrahydro-imidazoazepinol CSN5i-1a (Fig. 1b) as a starting point for further optimization, because it also weakly inhibited cullin-1 (Cul1) deneddylation in HCT116 cells as seen in a qualitative western blot analysis (not shown).

In a first round of compound optimization we generated CSN5i-1b (Fig. 1b), which has a slightly improved potency in the biochemical assay (Table 1) and could be co-crystallized with recombinant CSN5. Even though isolated CSN5 is not proteolytically active by itself, its active site appears to be largely intact as seen with the related protease AMSHLP^41^. We therefore speculated that the binding mode of CSN5i-1b to CSN5 resembles, to a significant degree, that of CSN5i-1b to the active CSN complex. The co-crystal structure revealed the imidazole of CSN5i-1b to coordinate the catalytic Zn^2+^-ion in a monodentate manner and the nitrile to point towards the putative substrate binding cleft (Fig. 1c, red arrow). In addition to the metal coordination, the ligand interacts with the protein predominantly via van der Waals interactions (Supplementary Fig. 1). A weak additional electron density peak in the putative substrate binding cleft close to the ligand nitrile was interpreted as methylpentandiol (MPD), a component of the crystallization buffer. Replacing the nitrile of CSN5i-1b with substituted aminophenyl groups led to compounds that extend into this cleft, for example, CSN5i-2 (Fig. 1b), replacing the MPD molecule, and possess significantly improved potency in biochemical and cellular assays (Table 1 and Supplementary Fig. 2). For further optimization, we focused on improving the pharmacokinetic properties of the compound by replacing the hydrophobic aminophenyl group with more polar heterocyclic moieties. The final compound, CSN5i-3 (Fig. 1b), inhibits CSN-catalysed Cul1 deneddylation with an IC_50_ value of 5.8 nM and shows a good pharmacokinetic profile (Fig. 1d and Tables 1 and 2).

The crystal structure revealed that the compound retained the binding mode of CSN5i-1b with the substituted pyrazole moiety extending into the hydrophobic channel displacing the buffer molecule. The higher binding affinity seems to be mainly driven by additional hydrophobic interactions with one additional putative hydrogen bond formed between the side chain of Thr154 and the hydrogen atom of the ligand's amide bond. Related JAMM domain containing proteases such as AMSHLP (ref. 41) and RPN11 (ref. 42), and a panel of other metalloproteases were not or only weakly inhibited by the compound, indicating its excellent selectivity (Table 1). Treatment of cells with CSN5i-3 resulted in the accumulation of neddylated Cul1, similar to the effect seen on CSN5 depletion by small interfering RNA (siRNA)^31^ (Fig. 1e). Moreover, inhibition of CSN5 correlated well with accumulation of neddylated Cul1, and the latter with cell proliferation (see below), for a large number of derivatives of the tetrahydro-imidazoazepinol chemotype (Supplementary Fig. 2), strongly indicating a direct link between CSN5 inhibition and the observed cellular effects. Thus, CSN5i-3 is a potent and selective inhibitor of CSN-catalysed deneddylation of NEDD8-modified CRLs.

### Effect of CSN5 inhibition on CRL substrates and SRMs

**Figure 2:**
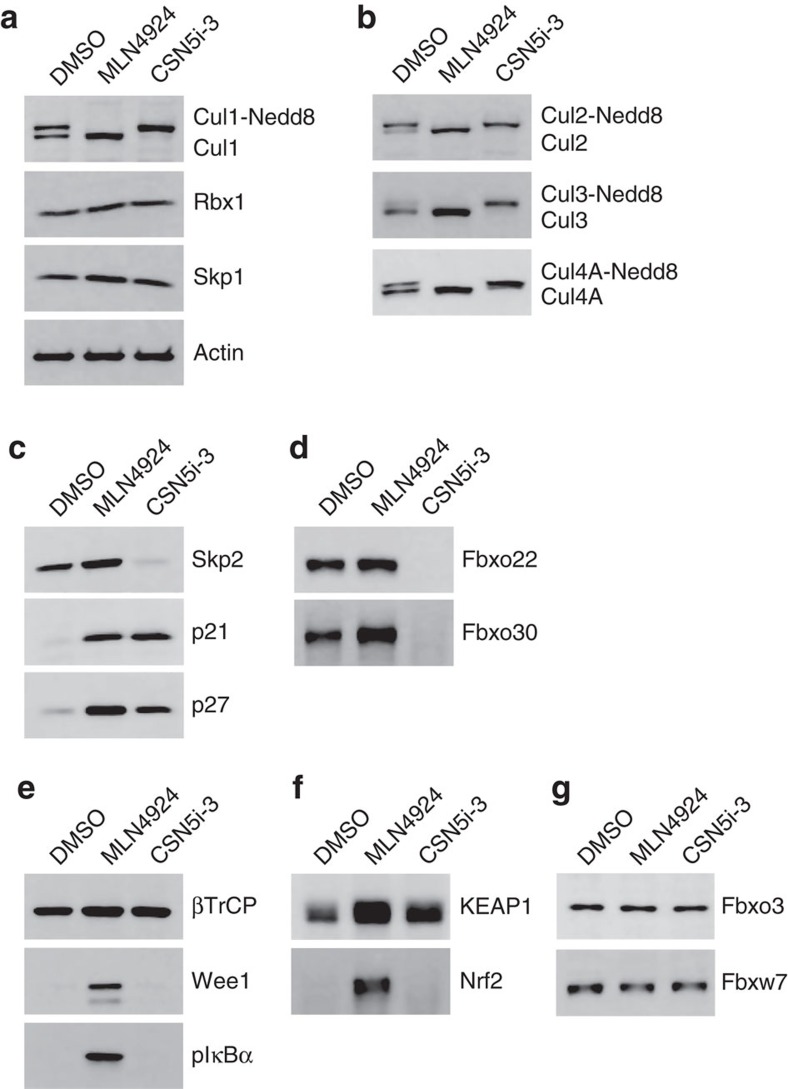
CSN5 inhibition results in the inactivation of a subset of CRLs and the stabilization of their substrates. Effect on CRL components and substrates after treatment of HCT116 cells with either 1 μM MLN4924 or 1 μM CSN5i-3. (**a**) Immunoblottings for Cul1 and its CRL components Rbx and Skp1; (**b**) for cullins Cul2, Cul3 and Cul4A; (**c**) for F-box protein Skp2 and the SCF^Skp2^ substrates p21 and p27; (**d**) for F-box proteins Fbxo22 and Fbxo30; (**e**) for F-box protein βTrCP and the SCF^βTrCP^ substrates Wee1 and pIκBα; (**f**) for the adaptor KEAP1 and the Cul3^KEAP1^ substrate Nrf2; (**g**) for F-box proteins Fbxw7 and Fbxo3.

We first investigated the effect of CSN5 inhibitors on the activation of CRLs in cultured tumour cells and analysed the consequences for CRL substrate turnover. HCT116 cells were treated with either CSN5i-3 or, for comparison, with the NAE1 inhibitor MLN4924 (refs 17, 43). The latter inhibitor, which is currently under investigation in phase II clinical trials as anti-cancer agent^17^,^43^, prevents the neddylation of CRLs and traps them in an inactive state (Figs 1a and 2a,b). As a result, the respective CRL substrates cannot be ubiquitinated and are protected from degradation by the proteasome (Fig. 2c,e,f). Moreover, SRMs, which are often auto-ubiquitinated by their respective CRL^44^,^45^,^46^, are also protected from degradation in the presence of MLN4924 (Fig. 2c–g). In contrast, treatment with CSN5i-3 resulted in the quantitative accumulation of cullins in the neddylated, active state (shown for Cul1, -2, -3 and -4A; Fig. 2a,b) and led to the degradation of SRMs including Skp2, Fbxo22 and Fbxo30 (Fig. 2c,d). Degradation of Skp2 was prevented by pre-incubation with proteasome inhibitors and pre-incubation with MLN4924 prevented Cul1 neddylation as well as Skp2 degradation (Supplementary Fig. 3a), probably indicating an auto-ubiquitination induced mechanism for CRL inactivation (Supplementary Fig. 3b). The substrates of these CRLs were also protected from degradation as shown for the SCF^Skp2^ substrates p21 and p27 (Fig. 2c). MLN4924 and CSN5i-3, therefore, can induce a very similar effect on CRL substrates, albeit through different and opposing mechanisms.

However, not all SRMs were prone to degradation upon CSN5 inhibition. Unlike Skp2, Fbxo22 and Fbxo30, levels of βTrCP, KEAP1, Fbxo3 and Fbxw7 remained stable on treatment of HCT116 cells with CSN5i-3 (Fig. 2e–g), and thus the substrates of the corresponding CRLs are affected differently by the two inhibitors. As shown for the SCF^βTrCP^ substrates Wee1 and pIκB, and the Cul3^KEAP1^ substrate Nrf2, treatment with the NAE1 inhibitor prevented the degradation of Wee1, pIκB and Nrf2, and resulted in their accumulation, whereas treatment with CSN5i-3 did not affect their degradation, which proceeded as in the absence of inhibitor (Fig. 2e,f). This result is consistent with the different mechanistic effects of the two inhibitors with MLN4924 trapping CRLs in the inactive state and CSN5i-3 locking CRLs in the active conformation. Collectively, our data show that the consequences of CSN5 inhibition are distinct from those of NAE1 inhibition (for additional differently affected proteins, see Supplementary Fig. 3c) and indicate that not all SRMs are equally susceptible to CRL-induced auto-ubiquitination and proteasomal degradation.

### CSN5 blockade exhibits a differentiating impact on cancer cells

**Figure 3:**
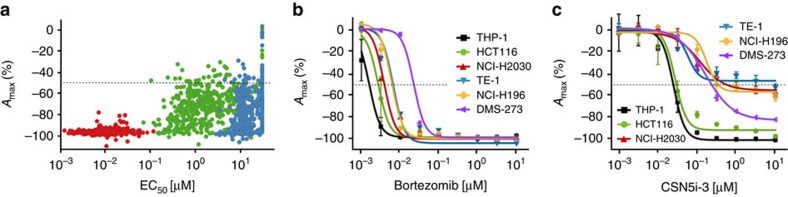
CSN5 inhibition exhibits a differentiating effect on the viability of cancer cells. (**a**) Concentration dependent effects of bortezomib (red), CSN5i-2 (green) and CSN5i-2e (blue) on a panel of cancer cell lines. Shown are the high-concentration effect level (*A*_max_) as derived from the sigmoidal fit of the individual dose–response curves and the corresponding transitional concentration values (EC_50_). (**b**,**c**) Individual dose response curves of bortezomib and CSN5i-3-treated cell lines (*n*=2, ±s.d.).

Next, we explored the effect of CSN5 inhibition on the viability of cancer cells. To obtain a comprehensive picture, we generated dose–response curves with a CSN5 inhibitor across more than 500 cancer cell lines in a previously described standardized setting^47^ (Supplementary Data 1). For the study we used CSN5i-2, a CSN5 inhibitor with an almost identical profile to CSN5i-3, but limiting pharmacokinetic parameters, and its poorly active *R,R*-enantiomer CSN5i-2e as a negative control to exclude off-target effects (Fig. 1b, Tables 1 and 2, and Supplementary Fig. 4a). As a benchmark we employed the proteasome inhibitor bortezomib, which is used in the clinic for the treatment of a variety of cancers^9^,^12^. Bortezomib was universally lethal and provoked cell death (*A*_max_ of −95% to −100%) across all cell lines (Fig. 3a). In contrast, CSN5i-2 was much more variable in its effects on the cell lines tested. Although the compound was cytotoxic to some cell lines to a similar degree as bortezomib (*A*_max_ ≤−80%), other cell lines appeared less sensitive to CSN5 inhibition with only a 40–60% maximal loss of viability (Fig. 3a). The *R,R*-enantiomer CSN5i-2e showed a similar variability in *A*_max_ values, yet a much higher compound concentration was required to have an impact on cell growth, reflecting its weaker potency towards CSN5 and excluding major off-target effects of our compounds. Interestingly, conducting analogous dose–response experiments with MLN4924 resulted in a similar overall pattern of cell viability across the panel of cancer cell lines as CSN5i-2 (Supplementary Fig. 4c).

For a selection of cell lines from the cancer cell line panel, we confirmed the differentiating effect of CSN5 inhibition on cell viability using CSN5i-3. Although bortezomib was again cytotoxic for all cell lines tested (*A*_max_ ≤−95%; Fig. 3b), treatment with CSN5i-3 resulted in a differential cell viability pattern as observed with CSN5i-2. To some cell lines, for example, THP-1 and HCT116, the presence of CSN5i-3 was cytotoxic (*A*_max_ ≤−95%), whereas to others, for example, NCI-H2030 and TE-1, the CSN5 inhibitor only led to cytostatic effects (*A*_max_ of about −50%; Fig. 3c). These cell line-specific effects were not due to a different degree of CSN5 inhibition, as CSN5i-3 effectively inhibited Cul1 deneddylation and promoted Skp2 degradation in the cell lines tested, including those in which the CSN5 inhibitor only showed a cytostatic effect (Supplementary Fig. 4b). Therefore, these results indicate a varying dependency of cancer cell lines on CSN5 activity and clearly differentiate CSN5 from proteasome inhibition.

### CSN5i-3 inhibits growth of a human xenograft *in vivo*

**Figure 4:**
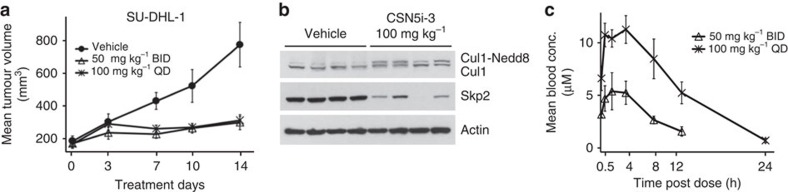
CSN5i-3 inhibits tumour growth of a human xenograft. (**a**) SU-DHL-1 xenografts were grown in SCID-bg mice and dosed by oral administration with either vehicle control or CSN5i-3 at the indicated doses and schedules. Mean tumour volumes are shown±s.e.m. (*n*=4; *P*<0.05). (**b**) Immunoblotting for Cul1 and Skp2 of tumours excised at the end of treatment. (**c**) Pharmacokinetics of CSN5i-3 post second to last dose. Mean blood concentrations are shown±s.e.m. (*n*=4).

When correlating sensitivity to CSN5 inhibition with the genetic background of the tested cancer cell lines^47^, we found that cell lines with CDK6 amplification (JHH-4, NOMO-1, OCI-M1 and SU-DHL-6) were highly sensitive and reached *A*_max_ values of ≤−95%. It is conceivable that the higher sensitivity to CSN5 inhibition of the above cell lines may be explained by the proposed orphan role of the F-box protein Fbxo7 as an enhancer of CDK6 activity^48^,^49^. Accordingly, Fbxo7 was depleted in, for example, CSN5i-3-treated NOMO-1 cells (Supplementary Fig. 5a). Intrigued by these findings, we selected the anaplastic large-cell lymphoma-derived cell line SU-DHL-1 (ref. 50) for an evaluation of the anti-tumour potential of CSN5i-3 in a xenograft model. For this cell line, a 7q22 amplification combined with CDK6 overexpression was reported^51^. SU-DHL-1 cells were indeed highly sensitive to treatment with CSN5i-3 *in vitro* with an EC_50_ value of 0.013 μM and *A*_max_ ≤−95%, and Fbxo7 levels were reduced on CSN5 inhibition (Supplementary Fig. 5b). Furthermore, treatment with CSN5i-3 triggered the formation of cleaved PARP and cleaved caspase 3 indicative of apoptosis induction (Supplementary Fig. 5c). For the *in vivo* study, subcutaneous SU-DHL-1 xenografts were grown in SCID-bg mice and CSN5i-3 was administered orally at doses of 50 mg kg^−1^ BID and 100 mg kg^−1^ QD over a period of 14 days. Tumour stasis was achieved with both dosing schemes (Fig. 4a) and inhibition of tumour growth calculated at the last day of treatment reached a T/C value of 22% (tumour volume of treated versus control samples) for the 50 mg kg^−1^ BID dose and a T/C value of 25% for the 100 mg kg^−1^ QD dose. Tumours were excised at the end of the study and the effect of compound treatment assessed on the pharmacodynamics markers, neddylated Cul1 and Skp2. In line with the anti-tumour efficacy, Cul1 deneddylation was inhibited and Skp2 levels were reduced in the tumours of the treated mice but not in those from vehicle controls (Fig. 4b). A dose-linear exposure was achieved with CSN5i-3 (Fig. 4c) and, based on body weight, both doses were well tolerated (Supplementary Fig. 5d). Based on the CSN5i-3 exposures that were significantly above the EC_50_ value for inhibition of cell proliferation and between 5 and 10 μM at peak and 1 and 2 μM at trough, we assume full inhibition of CSN5 over the time course of the experiment.

## Discussion

CSN5i-3 is a potent, selective and orally available inhibitor of the proteolytic subunit CSN5 of the CSN complex. It provides a novel modality by which the UPS can be modulated and differs in its effect from inhibitors of the proteasome. The CSN5 inhibitor interferes with the UPS in a unique manner by enforcing the auto-inactivation of only a subset of CRLs. Therefore, its impact on the UPS is significantly more restricted than that of a proteasome inhibitor, which translated into a strongly differentiating effect on cell growth and cell viability as compared to the treatment with bortezomib. Despite the broad impact on the UPS, bortezomib is successfully used in the clinic for the treatment of multiple myeloma and mantle cell lymphoma^52^,^53^. Expansion into other cancer indications is, however, hampered by its limited therapeutic window, which is likely to be due to its ubiquitous cytotoxic effects^11^,^12^. Based on our results, we expect a CSN5 inhibitor to potentially have a larger therapeutic window and to provide new therapeutic opportunities for a number of different cancers.

Our data show that the impact of a CSN5 inhibitor on the UPS is, to some degree, comparable with that of the more recently described NAE1 inhibitor MLN4924 (ref. 17). Promising results have been obtained with this drug candidate in preclinical experiments with various tumour cell lines, as well as with xenograft and transgenic rodent models, and multiple Phase I clinical trials are ongoing^43^. MLN4924 disturbs the UPS less broadly than the proteasome inhibitors, but still affects a wide range of cellular processes, as it prevents the activation of all CRLs and effects other proteins that are regulated by NEDD8 conjugation^54^. The biology of the latter is largely unexplored. The ongoing clinical trials will show whether the more specific impact on the UPS will indeed translate into a good therapeutic window in the clinic. Similarly, a CSN5 inhibitor, which affects only a subset of CRLs as revealed in our study, also modulates the UPS in a more specific manner than proteasome inhibitors. CSN5i-3 had a significantly more differentiating impact on cancer cell lines than bortezomib and thus might have a good prospect for an advantageous clinical profile. However, as we do not yet fully understand the differential dependency of cell lines on CSN5 and how these differences are based on genetic or epigenetic differences, comprehensive studies addressing the effects of CSN5i-3 on oncogenic and tumour suppressive CRLs are critical to facilitate the selection of cancer indications best suited for the treatment with a CSN5 inhibitor. In addition, through further comparative studies it will be interesting to learn more about the similarities and differences of MLN4924 and CSN5i-3 in terms of anti-tumour efficacy and overall clinical profile.

In addition to its therapeutic potential, CSN5i-3 might serve as an important research tool to further study the biology of CRLs and their regulation. For example, its use may facilitate the establishment of a comprehensive overview of SRMs that are regulated by auto-ubiquitination. Moreover, it further enables studying the consequences of modulating the UPS at the level of CRL assembly and disassembly on the regulation of specific molecular pathways and cellular processes.

To the best of our knowledge, CSN5i-3 is the first potent inhibitor described for the CSN and is the first example of an inhibitor of a JAMM domain-containing DUB enzyme. Our work suggests that this family of DUBs is amenable to the discovery of small molecular weight drugs. The JAMM domain-containing DUB family^29^ may include additional drug targets and the discovery of CSN5 inhibitors can help to find therapeutically relevant antagonists for other family members. One such as yet unexplored drug target might be BRCC36, which, similar to CSN5, acts in the context of an oligomeric complex^55^. In the BRCC36 isopeptidase complex, BRCC36 promotes interferon-dependent responses by deubiquitinating and stabilizing the type I interferon receptor^56^. Moreover, it acts as an activator of the NLRP3 inflammasome by deubiquitinating NLRP3 (ref. 57), thus suggesting its relevance as a potential target for the treatment of autoimmune diseases.

## Methods

### Synthesis of CSN5 inhibitors

Reaction schemes for the synthesis of CSN5 inhibitors are illustrated in Supplementary Fig. 6. All commercial reagents and anhydrous solvents were purchased and used without further purification, unless otherwise specified (Supplementary Fig. 6). Reactions were monitored by thin-layer chromatography (TLC), using silica gel F_254_ glass plates (5 × 10 cm) or by ultra performance-liquid chromatography–mass spectrometry (UPLC/MS) or HPLC with ultraviolet detection at 214, 220 or 254 nm. Purification was accomplished by flash chromatography using a Combi Flash R*f* 200 instrument (Teledyne Isco) and RediSep prepacked silica gel columns. All compounds submitted for biological testing were purified by preparative reversed-phase HPLC using either a Gilson HPLC or a Waters HPLC/MS system using a Waters XBridge Prep C18 OBD column (5 μm, 30 × 100 mm). Pure products were lyophilized from acetonitrile (ACN)/H_2_O. Compound purity was determined by UPLC/MS and HPLC as described below. Analytical UPLC/MS analysis was performed on a Waters Acquity UPLC single-stage quadrupole MS (Waters) with electrospray ionization in alternating positive and negative mode. Mass scan range was from 100 to –1,200 Da in a scan time of 0.3 s and recorded in centroid mode. Liquid chromatography was performed on an Acquity HSS T3 C18 column (1.8 μm, 2.1 × 50 mm) at 60 °C. Solvent A: 0.05% HCOOH, 3.75 mM ammonium acetate in H_2_O. Solvent B: 0.04% HCOOH in ACN. Flow rate=1.0 ml min^−1^. Gradient: 5%→98% B in 2 min. Analytical HPLC was performed on an Agilent Series 1200 instrument using a X-Bridge C18 column (2.5 μm, 3 × 50 mm; Waters) at 40 °C. Solvent A: 0.1% trifluoroacetic acid (TFA) in H_2_O. Solvent B: 0.1% TFA in ACN. Flow rate=1.4 ml min^−1^. Gradient: 10%→98% B in 8.6 min, 98%→98% B in 1.4 min. ^1^H-NMR spectra were recorded on a spectrometer (400 or 600 MHz; Bruker).

*Ethyl 4-(1-trityl-1H-imidazol-4-yl)butanoate **3***. To a suspension of 4-iodo-1-trityl-1*H*-imidazole (**2**) (250 g, 573 mmol) in dry THF (1.2 l) was added PdCl_2_(dppf). CH_2_Cl_2_ (46.8 g, 57.3 mmol) under an argon atmosphere. The reaction mixture was cooled to 0 °C and a solution of (4-ethoxy-4-oxobutyl)zinc(II)bromide (**1**) (0.5 M in THF) (1.72 l, 860 mmol) was added dropwise. Subsequently, the reaction mixture was stirred at 70 °C for 1.5 h. After cooling to ambient temperature, the reaction was quenched with H_2_O and filtered through a pad of celite. The filtrate was concentrated, the residue was poured into saturated NaHCO_3_ solution and extracted with EtOAc (3 ×). The combined organic layers were washed with H_2_O and brine, dried over Na_2_SO_4_, filtered, concentrated and the crude residue was purified by flash column chromatography eluting with a gradient of 10 to 100% EtOAc in hexanes to afford 181.4 g (73%) ethyl 4-(1-trityl-1H-imidazol-4-yl)butanoate (**3**). UPLC/MS: *R*_t_=1.10 min, 425.3 [M+H]^+^; ^1^H-NMR (400 MHz, DMSO-*d*_6_) *δ* (p.p.m.): 7.46–7.33 (m, 9H), 7.24 (s, 1H), 7.13–7.03 (m, 6H), 6.54 (s, 1H), 4.06–3.98 (q, *J*=8 Hz, 2H), 2.46–2.4 (m, 2H), 2.3–2.21 (m, 2H), 1.82–1.72 (m, 2H), 1.14 (t, *J*=8 Hz, 3H).

*6-Methyl-[1,1′-biphenyl]-3-carbonitrile **6***. To a solution of 3-bromo-4-methylbenzonitrile (**4**) (10 g, 51.0 mmol) in dimethylformamide (DMF)/H_2_O (1:1, 170 ml) were added phenylboronic acid (**5**) (9.33 g, 77 mmol), Cs_2_CO_3_ (24.93 g, 77 mmol) and PdCl_2_(dppf).CH_2_Cl_2_ (4.17 g, 5.10 mmol) under an argon atmosphere. The reaction was heated at 90 °C for 14 h. After cooling to ambient temperature, the reaction was diluted with EtOAc, filtered through a pad of celite and the filtrate was washed with H_2_O. The organic layer was dried and concentrated. The crude residue was purified by flash column chromatography eluting with hexanes during 20 min then with a gradient from 0 to 10% EtOAc over a period of 30 min to afford 9.62 g (97%) 6-methyl-[1,1′-biphenyl]-3-carbonitrile (**6**). TLC (hexanes/EtOAc, 9/1 v/v) *R*_F_=0.43. UPLC/MS: *R*_t_=1.20 min, 211 [M+NH_4_]^+^, HPLC: *R*_t_ =2.57 min; ^1^H-NMR (400 MHz, DMSO-*d*_6_) *δ* (p.p.m.): 7.74 (dd, 1H, *J*=7.9, 1.8 Hz), 7.65 (d, 1H, *J*=1.8 Hz), 7.59–7.32 (m, 6H), 2.30 (s, 3H).

*6-(Bromomethyl)-[1,1′-biphenyl]-3-carbonitrile **7***. A solution of 6-methyl-[1,1'-biphenyl]-3-carbonitrile (**6**) (9.62 g, 49.8 mmol), NBS (10.63 g, 59.7 mmol) and AIBN (0.817 g, 4.98 mmol) in CCl_4_ (166 ml) was heated at 80 °C for 15 h. After cooling to ambient temperature, the reaction mixture was poured into H_2_O and extracted with dichloromethane (DCM) (3 ×). The combined organic phase was dried, filtered, concentrated and the residue was purified by flash column chromatography eluting with a gradient of 0 to 5% EtOAc in hexanes to afford 13.2 g (68%) 6-(bromomethyl)-[1,1′-biphenyl]-3-carbonitrile (**7**). TLC (hexanes/EtOAc, 9/1 v/v) *R*_F_=0.39; UPLC/MS: *R*_t_=1.21 min, 290 [M+NH_4_]^+^; HPLC: *R*_t_=2.64 min; ^1^H-NMR (400 MHz, DMSO-*d*_6_) *δ* (p.p.m.): 7.92–7.70 (m, 3H), 7.57–7.42 (m, 5H), 4.62 (s, 2H).

*Ethyl 4-(1-((5-cyano-[1,1′-biphenyl]-2-yl)methyl)-1H-imidazol-5-yl)butanoate **8***. To a solution of ethyl 4-(1-trityl-1H-imidazol-4-yl)butanoate (**3**) (8.97 g, 21.13 mmol) in acetonitrile (220 ml) was added 6-(bromomethyl)-[1,1′-biphenyl]-3-carbonitrile (**7**) (5 g, 18.37 mmol). The reaction was stirred at ambient temperature for 15 h. Next, the reaction mixture was concentrated, the residue dissolved in MeOH (147 ml) and stirred at 70 °C for 3 h. After cooling to ambient temperature, the reaction mixture was concentrated and the residue was purified by flash column chromatography eluting with a gradient of 0 to 10% EtOAc in hexanes to afford **8**. Subsequently, product **8** was dissolved in EtOAc and washed with saturated NaHCO_3_ solution (3 ×), the organic phase was dried and concentrated to yield 8.1 g, (94%) ethyl 4-(1-((5-cyano-[1,1′-biphenyl]-2-yl)methyl)-1H-imidazol-5-yl)butanoate (**8**). TLC (hexanes/EtOAc, 9/1 v/v) *R*_F_=0.45; UPLC/MS: *R*_t_=0.81 min, 375.0 [M+H]^+^; ^1^H-NMR (400 MHz, DMSO-*d*_6_) *δ* (p.p.m.): 8.10 (s, 1H), 7.90–7.75 (m, 2H), 7.58–7.38 (m, 5H), 7.10–6.99 (m, 2H), 5.28 (s, 2H), 3.99 (q, *J*=8 Hz, 2H), 2.18 (t, 2H), 2.16 (t, *J* =7.2 Hz, 2H), 1.56 (m, *J*=7.2 Hz, 2H), 1.14 (t, *J*=8 Hz, 3H).

*6-(6-Oxo-6,7,8,9-tetrahydro-5H-imidazo[1,5-a]azepin-5-yl)-[1,1′-biphenyl]-3-carbonitrile **9***. A solution of ethyl 4-(1-((5-cyano-[1,1′-biphenyl]-2-yl)methyl)-1H-imidazol-5-yl)butanoate (**8**) (7.67 g, 16.43 mmol) in dry THF (41 ml) was degased with argon during 20 min. To this solution was added dropwise over a period of 10 min a solution of potassium *tert*-butoxide sublimed grade (4.06 g, 36.1 mmol) in dry THF (41.1 ml) under an argon atmosphere. The reaction was stirred for 3 h at ambient temperature under argon atmosphere. The reaction was quenched with aqueous saturated NH_4_Cl solution and extracted with DCM (3 ×). The organic phase was dried, concentrated and the residue purified by flash column chromatography eluting with a gradient of 0 to 10% MeOH in DCM to afford 4.5 g (84%) 6-(6-oxo-6,7,8,9-tetrahydro-5H-imidazo[1,5-a]azepin-5-yl)-[1,1′-biphenyl]-3-carbonitrile (**9**). TLC (hexanes/EtOAc, 9/1 v/v) *R*_F_=0.46; UPLC/MS: *R*_t_=0.72 min, 328.0 [M+H]^+^; ^1^H-NMR (400 MHz, DMSO-*d*_6_) *δ* (p.p.m.): 7.83–7.81 (m, 1H), 7.76 (s, 1H), 7.55 (s, 1H), 7.5–7.01 (m, 5H), 6.86 (m, 2H), 6.29 (s, 1H), 2.43–2.34 (m, 4H), 1.92–1.81 (tt, *J*=7.1 Hz, 2 H).

*Cis-6-(6-Hydroxy-6,7,8,9-tetrahydro-5H-imidazo[1,5-a]azepin-5-yl)-[1,1′-biphenyl]-3-carbonitrile **CSN5i-1a***. To a solution of 6-(6-oxo-6,7,8,9-tetrahydro-5H-imidazo[1,5-a]azepin-5-yl)-[1,1′-biphenyl]-3-carbonitrile (**9**) (3.72 g, 11.38 mmol) in MeOH (228 ml) at 0 °C was added NaBH_4_ (0.452 g, 11.95 mmol) in portions. The reaction was stirred at 0 °C for 1 h and subsequently quenched with H_2_O. MeOH was evaporated and the aqueous phase was extracted with EtOAc (3 ×) and DCM (2 ×). The combined organic layers were dried, concentrated and the residue was purified by flash column chromatography eluting with a gradient of 0 to 10% MeOH in DCM, to afford 3.35 g (94%) **CSN5i-1a.** TLC (DCM/MeOH, 9/1 v/v) *R*_F_= 0.33; UPLC/MS: *R*_t_=0.70 min, 330.1 [M+H]^+^; ^1^H-NMR (400 MHz, DMSO-*d*_6_) *δ* (p.p.m.): 8.45 (d, *J*=8.2 Hz,1H), 8.05 (dd, *J*=8.2, 1.7 Hz, 1H), 7.82 (d, *J*=1.7 Hz,1H), 7.44–7.30 (q, *J*=1.7, 6.1 Hz, 3H), 7.00 (d, *J*=6.5 Hz, 2H), 6.67 (s, 1H), 6.62 (s, 1H), 5.19 (d, *J*=4.3 Hz, 1H), 5.04 (s, 1H), 4.19 (s, 1H), 2.82–2.75 (d, J=16.4 Hz, 1H), 2.02–1.06 (d, *J*=22.7 Hz, 2H), 1.68–1.46 (m, 3H).

*6-(6-Hydroxy-6,7,8,9-tetrahydro-5H-imidazo[1,5-a]azepin-5-yl)-[1,1′-biphenyl]-3-carboxylic acid **10***. To a solution of 6-(6-hydroxy-6,7,8,9-tetrahydro-5*H*-imidazo[1,5-a]azepin-5-yl)-[1,1′-biphenyl]-3-carbonitrile (**CSN5i-1a**) (6 g, 18.22 mmol) in THF/MeOH (1:1; 180 ml) was added a solution of NaOH (10.92 g, 274 mmol) in H_2_O (54 ml). The reaction mixture was stirred at 60 °C for 24 h. After cooling to 0 °C the pH of the reaction mixture was adjusted to 4.5 by dropwise adding TFA. The reaction was concentrated and the residue was purified by preparative HPLC, solvent A: H_2_O+0.1% TFA, solvent B: ACN+0.1% TFA, gradient: 5→100% B in 20 min, flow rate=40 ml min^−1^. Pure fractions were combined and lyophilized to afford 6.6 g (78%) of the title compound **10**. UPLC/MS: *R*_t_=0.61 min, 349.2 [M+H]^+^; HPLC: *R*_t_=1.52 min. ^1^H-NMR (400 MHz, DMSO-*d*_6_) *δ* (p.p.m.): 13.26 (br s, 1H), 8.22 (m, 3H), 7.84 (d, J=1.71 Hz, 1H), 7.39–7.35 (m, 4H), 7.15 (br s, 2H), 5.52 (s, 1H), 5.51 (m, 1H), 4.50 (br m, 1H), 2.90 (br dd, *J*=14.91, 5.84 Hz, 1H), 2.21–1.40 (m, 5H).

*Tert-butyl((6-((5R,6R)-6-hydroxy-6,7,8,9-tetrahydro-5H-imidazo[1,5-a]azepin-5-yl)-[1,1′-biphenyl]-3-yl)carbamate **11** and tert-butyl((6-((5S,6S)-6-hydroxy-6,7,8,9-tetrahydro-5H-imidazo[1,5-a]azepin-5-yl)-[1,1′-biphenyl]-3-yl)carbamate **12***. To a solution of 6-(6-hydroxy-6,7,8,9-tetrahydro-5*H*-imidazo[1,5-a]azepin-5-yl)-[1,1′-biphenyl]-3-carboxylic acid (**10**) (5.44 g, 11.76 mmol) and triethylamine (TEA) (4.1 ml, 29.4 mmol) in THF (39.2 ml) was added diphenyl phosphoryl azide (DPPA) (2.8 ml, 12.9 mmol). The reaction mixture was stirred at ambient temperature overnight. The reaction was concentrated and the residue dissolved in dry *t*-butanol (39.2 ml) under an argon atmosphere. The reaction was heated at 80 °C for 3 h. After cooling to ambient temperature, the reaction was concentrated, the residue dissolved in EtOAc and washed with saturated NaHCO_3_ solution. The aqueous layer was extracted with EtOAc (3 ×). The combined organic layers were dried, filtered, concentrated and purified by flash column chromatography eluting with a gradient of 0 to 10% MeOH in DCM to afford 3.38 g (68%) tert-butyl(6-(6-hydroxy-6,7,8,9-tetrahydro-5H-imidazo[1,5-a]azepin-5-yl)-[1,1′-biphenyl]-3-yl)carbamate. *Tert-*butyl(6-(6-hydroxy-6,7,8,9-tetrahydro-5*H*-imidazo[1,5-a]azepin-5-yl)-[1,1′ biphenyl]-3-yl)carbamate was separated into the enantiomers **11** and **12** by preparative chiral HPLC chromatography on a Chiralpak IC column (20 μm, 500 × 50 mm) eluting with heptane:DCM:ethanol (80:15:5, v/v/v)+0.05% diethylamine (DEA), flow rate 100 ml min^−1^, to afford 1.35 g (45%) tert-butyl((6-((5R,6R)-6-hydroxy-6,7,8,9-tetrahydro-5*H*-imidazo[1,5-a]azepin-5-yl)-[1,1′-biphenyl]-3-yl)carbamate (**11**) and 1.4 g (47%) tert-butyl((6-((5 S,6 S)-6-hydroxy-6,7,8,9-tetrahydro-5*H*-imidazo[1,5-a]azepin-5-yl)-[1,1′-biphenyl]-3-yl)carbamate (**12**). UPLC/MS: *R*_t_=0.93 min, 420 [M+H]^+^; HPLC: *R*_t_=3.91 min;^1^H-NMR (400 MHz, DMSO-*d*_6_) *δ* (p.p.m.): 9.55 (s, 1H), 8.13 (d, *J*=8.6 Hz, 1H), 7.55 (dd, *J*=8.6, 2.4 Hz, 1H), 7.44 (d, *J*=2.2 Hz,1H), 7.40–7.24 (m, 3H), 7.01–6.85 (m, 2H), 6.73–6.51 (m, 2H), 5.00 (d, *J*=4.1 Hz, 1H), 4.89 (s, 1H), 4.13–4.08 (d, *J*=4.7 Hz, 1H), 2.79 (d, *J*=15.8 Hz, 1H), 2.08–1.83 (dd, *J*=33.0, 17.8 Hz, 2H), 1.54–1.45 (m, 3 H), 1.47 (s, 9H).

*(5S,6S)-5-(5-amino-[1,1′-biphenyl]-2-yl)-6,7,8,9-tetrahydro-5H-imidazo[1,5-a]azepin-6-ol **13***. To a solution of *tert*-butyl (6-((5 S,6 S)-6-hydroxy-6,7,8,9-tetrahydro-5H-imidazo[1,5-a]azepin-5-yl)-[1,1′-biphenyl]-3-yl)carbamate (**12**) (1.4 g, 3.34 mmol) in dioxane/MeOH (1:1), 16 ml) was added HCl (4 M) in dioxane (13.4 ml). The reaction mixture was stirred at ambient temperature for 4 h and concentrated. The residue was purified by preparative HPLC, solvent A: H_2_O+0.1% TFA, solvent B: ACN+0.1% TFA, gradient: 5→100% B in 20 min, flow rate=40 ml min^−1^. Pure fractions were combined, saturated NaHCO_3_ solution was added and the aqueous phase was extracted with DCM (5 ×). The combined organic layers were dried and concentrated to give 1.02 g (91%) of the title compound **13**. UPLC/MS: *R*_t_=0.64 min, 320 [M+H]^+^; HPLC: *R*_t_=1.26 min; ^1^H-NMR (400 MHz, DMSO-*d*_6_) *δ* (p.p.m.): 7.91 (d, *J*=8.46 Hz, 1H), 7.28 (m, 3H), 6.87 (m, 2H), 6.80–6.61 (m, 2H), 6.55 (m, 1H), 6.47 (d, *J*=2.42, 1H), 5.32 (s, 2H), 4.87 (m, 1H), 4.79 (s, 1H), 4.11 (m, 1H), 2.79 (br d, *J*=14.41 Hz, 1H), 2.06–1.82 (m, 2H), 1.68–1.37 (m, 3H).

*Ethyl 3-(difluoromethyl)-1-isopropyl-1H-pyrazole-5-carboxylate **15***. To a solution of ethyl 3-(difluoromethyl)-1H-pyrazole-5-carboxylate (**14**) (1 g, 5.2 mmol) in dry dimethylformamide (DMF) (20 ml) under an argon atmosphere was added K_2_CO_3_ (1.8 g, 13.15 mmol). The reaction was stirred at ambient temperature for 10 min then 2-iodopropane (550 μl, 5.2 mmol) was added and the mixture was stirred at 40 °C for 3 h. After cooling to ambient temperature, H_2_O was added and the mixture was extracted with EtOAc (3 ×). The combined organic phase was dried, concentrated and the residue was purified by flash column chromatography eluting with a gradient of 0 to 50% EtOAc in hexanes to afford 0.940 g (75%) **15**. TLC (hexanes/EtOAc, 8:2 v/v) *R*_F_=0.61; UPLC/MS: *R*_t_=1.13 min, 233.1 [M+H]^+^; HPLC: *R*_t_=2.47 min; ^1^H-NMR (400 MHz, DMSO-*d*_6_) *δ* (p.p.m.): 7.27–6.89 (m, 2H), 5.46 (m, *J*=6.8 Hz, 1H), 4.33 (q, *J*=7.9 Hz, 2H), 1.44 (dd, *J*=6.8 Hz, 6H), 1.33 (t, *J*=7.9 Hz 3H).

*3-(Difluoromethyl)-1-isopropyl-1H-pyrazole-5-carboxylic acid **16***. To a solution of ethyl 3-(difluoromethyl)-1-isopropyl-1H-pyrazole-5-carboxylate (**15**) (900 mg, 0.38 mmol) in THF/H_2_O/MeOH (1:1:1, 18 ml) was added LiOH.H_2_O (162 mg, 0.38 mmol). The reaction mixture was stirred at ambient temperature for 4 h and the reaction was concentrated. The residue was taken up in HCl (1 N) and extracted with EtOAc (3 ×). The combined organic layers were dried and concentrated. The title compound **16** 0.77 g (99%) was used without further purification. UPLC/MS: *R*_t_=0.72 min, 205 [M+H]^+^; HPLC: *R*_t_=1.78 min; ^1^H-NMR (400 MHz, DMSO-*d*_6_) *δ* (p.p.m.): 13.98 (br s, 1H), 7.32–6.79 (m, 2H), 5.52 (qq, *J*=6.9 Hz, 1H), 1.42 (d, *J*=6.9 Hz, 6H).

*3-(Difluoromethyl)-N-(6-((5S,6S)-6-hydroxy-6,7,8,9-tetrahydro-5H-imidazo[1,5-a]azepin-5-yl)-[1,1′-biphenyl]-3-yl)-1-isopropyl-1H-pyrazole-5-carboxamide **CSN5i-3***. To a solution of (5 S,6 S)-5-(5-amino-[1,1′-biphenyl]-2-yl)-6,7,8,9-tetrahydro-5H-imidazo[1,5-a]azepin-6-ol (**13**) (1.0 g, 3.13 mmol) in DCM (18 ml) was added 3-(difluoromethyl)-1-isopropyl-1*H*-pyrazole-5-carboxylic acid (**16**) (0.77 g, 3.76 mmol), N,N-diisopropylethylamine (DIPEA) (2.3 ml, 4 mmol) and propylphosphonic anhydride (T3P) (1.6 ml, 9.3 mmol). The reaction was stirred at ambient temperature for 2 h, subsequently concentrated and the residue was purified by preparative HPLC, solvent A: H_2_O+7.3 mM NH_4_OH, solvent B: ACN+7.3 mM NH_4_OH, gradient: 5→99% B in 12.5 min and 99% B maintained for 2.5 min, flow rate=45 ml min^−1^. Pure fractions were combined and lyophilized to afford 1.2 g (74%) **CSN5i-3**. UPLC/MS: *R*_t_=0.91 min, 506.4 [M+H]^+^; HPLC; *R*_t_=4.29 min. ^1^H-NMR (600 MHz, DMSO-*d*_6_) *δ* (p.p.m.): 10.59 (s, 1H), 8.28 (d, *J*=8.6 Hz, 1H), 7.90 (dd, *J*=8.6, 1.9 Hz, 1H), 7.80 (d, *J*=1.8 Hz, 1H), 7.41–7.24 (m, 4H), 7.10 (t, *J*=54.6 Hz, 1H), 6.98 (d, *J*=5.7 Hz, 2H), 6.73 (s, 1H), 6.61 (s, 1H), 5.44 (p, *J*=6.6 Hz, 1H), 5.14 (d, *J*=3.6 Hz, 1H), 4.99 (s, 1H), 4.20 (s, 1H), 2.82 (d, *J*=15.2 Hz, 1H), 2.03 (dt, *J*=14.2, 6.9 Hz, 1H), 2.00–1.89 (m, 1H), 1.66–1.49 (m, 3H),1.44 (d, *J*=6.6 Hz, 6H). HRMS (*m/z*) [M+H]^+^ calcd for C28H29N5O2F2 506.2362, found 506.2362.

*(5S,6S)-5-(2′-chloro-5-(((2-(methoxymethyl)phenyl)amino)methyl)-[1,1′-biphenyl]-2-yl)-6,7,8,9-tetrahydro-5H-imidazo[1,5-a]azepin-6-ol **CSN5i-2** and (5R,6R)-5-(2′-chloro-5-(((2-(methoxymethyl)phenyl)amino)methyl)-[1,1′-biphenyl]-2-yl)-6,7,8,9-tetrahydro-5H-imidazo[1,5-a]azepin-6-ol **CSN5i-2e.*** Compounds were prepared in analogy to the synthesis described for **CSN5i-3**. **CSN5i-2;** UPLC/MS: *R*_t_=0.97 and 1.00 min (atropisomers), 488 [M+H]^+^; HPLC: *R*_t_=4.10 and 4.27 min (atropisomers); ^1^H-NMR (400 MHz, DMSO-d_6_) *δ* (p.p.m.): 8.21 and 8.11 (d, *J*=8.16 Hz, 1H), 7.56 (m, 2H), 7.38 (m, 2H), 7.13 (m, 4H), 6.62 (m, 4H), 5.71 (m, 1H), 5.05 (m, 1H), 4.47–4.15 (m, 5H), 3.26 (s, 3H), 2.73 (m, 1H), 2.43 (m, 1H), 2.01–1.26 (m, 5H). HRMS (*m*/*z*) [M+H]^+^ calcd for C29H30ClN3O2 488.2099, found 488.2099. **CSN5i-2e**; UPLC/MS: *R*_t_=0.98 and 1.00 min (atropisomers), 488 [M+H]^+^; HPLC: *R*_t_=4.11 and 4.28 min (atropisomers); ^1^H-NMR (400 MHz, DMSO-d_6_) *δ* (p.p.m.): 8.21 and 8.11 (d, *J*=8.16 Hz, 1H), 7.56 (m, 2H), 7.39 (m, 2H), 7.13 (m, 4H), 6.76–6.52 (m, 4H), 5.73 (m, 1H), 5.06 (m, 1H), 4.67 (s, 1H), 4.48–4.25 (m, 4H), 3.25 (s, 3H), 2.74 (m, 1H), 2.16–1.84 (m, 2H), 1.59–1.25 (m, 3H). HRMS (m/z) [M+H]^+^ calcd for C29H30ClN3O2 488.2099, found 488.2097.

### Co-crystal structures of CSN5 with CSN5i-1b and CSN5i-3

Human CSN5(2–257) was expressed in *Escherichia coli* with an amino-terminal His_6_ tag and a Tobacco etch virus (TEV) protease cleavage site. In brief, expression from pET-28b vector in *E. coli* grown in lysogeny broth (LB) media was induced by the addition of 0.1 mM isopropyl-β-D-thiogalactoside at OD_600_ 0.5 and expression continued for 16 h at 20 °C. Pelleted cells were resuspended in 50 mM Tris-HCl pH 8, 200 mM NaCl and 20 U ml^−1^ Benzoase Nuclease (Merck), frozen and subsequently homogenized in the presence of protease inhibitors AEBSF and Complete EDTA-free inhibitor cocktail (Roche) using a French Press. Filtered cell lysates were purified on an IMAC column (10 ml bedvolume, Ni-NTA Superflow; Qiagen) attached to an Aekta Explorer FPLC system (GE Healthcare). CSN5(2–257) containing fractions were concentrated, treated with Tobacco etch virus (TEV) protease (Promega) and applied to a size-exclusion chromatography column (Superdex 75, HiLoad 26/60; GE Healthcare), and eluted in 25 mM MES-Na pH 6.0, 50 mM NaCl and 1 mM tris(2-carboxyethyl)phosphine (TCEP). The protein was concentrated 25 mg ml^−1^ and flash cooled in liquid nitrogen.

Crystals of CSN5(2–257) were obtained under several conditions using commercially available crystallization solutions, for example, JCSG-Core I suite condition F12, 0.1 M phosphate citrate pH 4.2 with 40% MPD (Molecular Dimensions), but only in the presence of ligand and additional amounts of zinc ions. They were subsequently improved by crystal seeding. In the final procedure, 0.3 μl of CSN5(2–257) at a concentration of 15 mg ml^−1^ in 50 mM NaCl, 50 mM MES/NaOH at pH 6.0, 1 mM TCEP, 1 mM CSN5i-1b and 10 μM ZnCl_2_ were mixed with 0.3 μl crystallization solution and 0.1 μl of seed solution in a 96-well Intelli crystallization plate (Hampton Research) and equilibrated at room temperature against 35% MPD, 0.2 M lithium sulfate and 0.1 M MES/NaOH at pH 6.0. For diffraction experiments, the crystals were mounted from mother liquor and flash-cooled without the addition of additional cryo-protectant. Diffraction data were collected at the Swiss Light Source beamline X10SA (Swiss Light Source, Paul Scherrer Institute, Switzerland) equipped with a Pilatus Pixel Detector (Dectris, Switzerland). The CSN5(2–257)-CSN5i-3 complex was obtained by using crystals grown in the presence of CSN5i-1a and subsequent back-soaked with 10 mM CSN5i-3 overnight. All crystallographic data were processed with XDS/XSCALE as part of the APRV package^58^. The CSN5(2–257)-CSN5i-1b structure was solved using phaser^59^ and 4f7o as search model^60^. Coot^61^ was used for visual inspection and models were refined with autoBuster (BUSTER version 2.11.5; Global Phasing Ltd). All structure figures were generated using pymol (The PyMOL Molecular Graphics System version 1.5.0.4; Schrödinger LLC). Data collection and structure refinement statistics are summarized in Supplementary Table 2.

### Protein expression and purification

CSN and Cul4A-Rbx1-DDB1-DDB2 complexes were expressed in Sf21 cells by co-infection of separate baculoviruses as previously described^30^. The cells were lysed by sonication, followed by centrifugation at 50,000 *g* for 50 min at 4 °C. Ni-sepharose FastFlow resin (GE Healthcare) was added to the cleared supernatant for batch purification. The eluted protein was dialysed against buffer A (20 mM Tris-HCl pH 8 and 1 mM dithiothreitol (DTT)) and applied to a Source Q ion-exchange chromatography column (GE Healthcare) equilibrated in buffer A. A NaCl gradient of 0–1 M NaCl was applied and protein containing fractions harvested, concentrated and stored at −80 °C before further use.

The purified Cul4A complex was next enzymatically modified with PT22-lablelled NEDD8. The required E1 and E2 enzymes for neddyation (UBA3/NEA1 complex and UBC12) were expressed in *E. coli* with N-terminal HRV 3C protease cleavable extensions (His_6_-ZZ-3C for NAE1, His_6_-thioredoxin-S tag-3C for UBC12 and His_6_-ZZ-3C-Cys for NEDD8 (ref. 40)). Proteins were affinity purified using Ni-sepharose FastFlow resin (GE Healthcare) followed by protease tag removal and size-exclusion chromatography in 50 mM Tris-HCl pH 7.4, 150 mM NaCl, 10% glycerol for UBA3/NAE1 and Cys-NEDD8, or 25 mM Tris-HCl pH 7.5, 50 mM NaCl, 5 mM MgCl_2_ and 0.25 mM DTT for UBC12. Cys-NEDD8 was next labelled in the dark under argon with PT-22 maleimide (AssayMetrics) in 50 mM Tris pH 7.5, 150 mM NaCl, 0.5 mM TCEP and 10% glycerol using a 5 × molar excess of PT22. For subsequent neddylation of the Cul4A complex, UBA3/NAE1, UBC12, PT22-labelled NEDD8 and Cul4A complex were mixed at 0.5, 0.5, 10 and 4 μM final concentration, respectively, in 50 mM Tris-HCl pH 7.5, 100 mM NaCl, 12.5 mM ATP, 0.5 mM DTT and 2.5 mM MgCl_2_. The solution was incubated for 2 h at room temperature and then purified on a HiLoad 16/60 Superdex 200 size-exclusion chromatography column equilibrated in 50 mM Tris pH 7.5, 100 mM NaCl, 0.05% CHAPS and 1 mM DTT.

### CSN5 activity assay

The potency of CSN5 inhibitors was determined in a CSN activity assay using changes in fluorescence polarization as the readout^30^. Reactions were carried out at room temperature in 384-well plates. Stock solutions (10 mM) of test compound in dimethyl sulfoxide (DMSO) were pre-diluted in 90% (v/v) DMSO/water to 101 times the desired assay concentration. Compound solution (0.25 μl) was then added per well, followed by the addition of 12.5 μl CSN solution (150 pM final assay concentration). After 1 h pre-incubation, the reaction was started by the addition of 12.5 μl PT22-Nedd8-Cul4A complex solution (150 nM final assay concentration) and allowed to progress for 1 h. The effect of compound on enzymatic activity was determined from the linear part of the progression curves by an end-point measurement of the fluorescence polarization at 590 nm after excitation at 540 nm using a PHERAstar plate reader (BMG LABTECH GmbH). IC_50_ values were calculated from the plot of percentage of inhibition versus inhibitor concentration using the non-linear regression analysis software Origin 7.5SR6 (OriginLab Corp.).

### Selectivity assays

All biochemical assays were performed at room temperature in 384-well plates in a total volume of 25.25 μl per well. Test compounds were dissolved in 90% (v/v) DMSO/water. For the assays, 250 nl of compound solution was added per well, followed by addition of 12.5 μl protease solution. After 70 min of pre-incubation at room temperature, the reactions were started by addition of 12.5 μl substrate solution and followed either by measuring fluorescence intensity (rhodamine 110-based substrates) or by fluorescence lifetime (PT14-based substrates). The effect of compound on enzymatic activity was obtained from the linear part of the progress curves and determined after 1 h (*t*=60 min). The final compound concentrations ranged from 100 μM to 1 nM.

*RPN11 (26S proteasome)*. Enzyme purchased from LifeSensors (catalogue number PS026); substrate: K48-linked tetra-ubiquitin chain coupled to a rhodamine 110 fluorophore purchased from LifeSensors (catalogue number NO13–158.01); enzyme concentration: 0.75 nM; substrate concentration: 100 nM; assay buffer: 20 mM Hepes at pH 7.4, 150 mM NaCl, 0.02% Tween-20, 0.5 mM TCEP, 0.05 mM MgCl_2_, 1 mM ATP and 2 μM ubiquitin aldehyde.

*Human AMSHLP*. Enzyme was expressed in and purified from *E. coli*; substrate: ubiquitin-rhodamine110-glycine was purchased from emp Biotech GmbH (Berlin, Germany; product number AS 09/12-A4); enzyme concentration: 4 nM; substrate concentration: 200 nM; assay buffer: 100 mM Tris/HCl at pH 7.5, 10 mM CaCl_2_, 100 mM NaCl, 10 μM ZnCl_2_, 0.005% (v/v) BRIJ 35 and 5 mM dithioerythritol (DTE).

*Matrix metalloproteinases*. Human MMP1 (Uniprot P03956), amino acids 1–469, expressed in and purified from stable transfected C127 cells; human MMP2 (Uniprot P08253), amino acids 1–660, expressed in and purified from insect cells (baculovirus expression system); human MMP8 (Uniprot P22894), amino acids 100–262, expressed in and purified from *E. coli*; human MMP9 (Uniprot P14780), amino acids 1–707, expressed in and purified from stable transfected 293 cells; human MMP12 (Uniprot P39900), amino acids 101–268, expressed in and purified from *E. coli*; human MMP13 (Uniprot P45452), amino acids 103–274, expressed in and purified from *E. coli*; human MMP14 (Uniprot P50281), amino acids 112–284, expressed in and purified from *E. coli*; human ADAM17 (Uniprot P78536), amino acids 1–670, expressed in and purified from insect cells (baculovirus expression system); substrate: Ac-C(PT14)-KPLGLWAR-NH2 purchased from Biosyntan (product number 8069); enzyme concentrations: 3 nM (MMP1), 1 nM (MMP2), 3 nM (MMP8), 0.1 nM (MMP9), 0.5 nM (MMP12), 0.13 nM (MMP13), 0.005 nM (MMP14), 30 nM (ADAM17); substrate concentration: 0.8 μM; assay buffer: 100 mM Tris, pH 7.4, 100 mM NaCl, 10 mM CaCl2, 10 μM ZnCl_2_, 0.075% (v/v) Brij35.

### Cell culture experiments

Cells were maintained in a humidified incubator at 37 °C and 5% CO_2_. HCT116 cells (ATCC #CCL-247) were grown in McCoy's 5A medium (GIBCO #16600–082) supplemented with 10% fetal bovine serum (FBS), TE-1 (RIKEN BioResource Center, #RCB1894), NCI-H2030 (ATCC #CRL-591), SU-DHL-1 (DMSZ #ACC 356), NOMO-1 (HSRRB #IFO50474) and NCIH196 cells (ATCC, #CRL-5823) in RPMI-1640 medium (GIBCO#61870–010) supplemented with 10% FBS, 10 mM HEPES, 1 mM sodium pyruvate and THP-1 cells (ATCC #TIB-202) with additional 0.05 mM 2-mercaptoethanol. DMS273 cells (ECACC #950628) were cultured in Waymouth's MB 752/1 medium (GIBCO#11220–035) supplemented with 10% FBS. All cells were negatively tested for mycoplasma contamination either by the vendor or in house. For treatment with compounds, sub-confluent cells were either treated with inhibitors at the indicated concentrations or mock treated with an equivalent amount of DMSO (final content 0.1%). If not stated otherwise, cells were harvested after incubation for 16 h and subjected to SDS–PAGE and western blotting. The RNA interference experiment was performed with CSN5 stealth siRNA (#HSS116986) and stealth negative control siRNA (#12935–400) (Invitrogen), which were transfected at 10 nM into HCT116 cells using HiperFect (Qiagen) according to the manufacturer's protocols. Seventy-two hours after transfection, cells were harvested and subjected to SDS–PAGE and western blotting (see Supplementary Fig. 7 for all western blottings performed).

For further analysis, cell lysates were prepared by direct lysis with 2 × Sample buffer (65.8 mM Tris-HCl, pH 6.8, 2.1% SDS, 26.3% glycerol, 0.01% bromophenol blue and 20 mM DTT). Protein lysates from tumour powder were prepared in RIPA buffer (50 mM Tris-HCl pH 8.0, 150 mM NaCl, 1% Triton X-100, 2 mM EDTA, 0.5% sodium deoxycholate and 0.1% SDS) supplemented with 10 mM *N*-ethylmaleimide, protease inhibitor and phosphatase inhibitor cocktails (Roche Diagnostics). Cells from longer compound incubations were also lysed in RIPA buffer, which allows the protein concentration to be determined by BCA Protein Assay Kit (Pierce). Proteins were separated by SDS–PAGE using 4–20% Tris-Glycine Gels (Invitrogen) and subsequently transferred to polyvinylidene difluoride membranes using a Trans-Blot Turbo System (BioRad). Membranes were subsequently blocked with 2% ECL Prime Blocking Reagent (GE Healthcare) in PBS 0.1% Tween-20 for 1 h. Primary antibodies were as follows: β-TrCP (1:1,000; #11984), Cul4A (1:1,000; #2699), pIκBα (Ser32, 1:1,000; #2859), p27 (1:1,000; #3688), Skp1 (1:1,000; #2156), Skp2 (1:1,000; #2652), Wee1 (1:1,000; #4936), NRF2 (1:1,000; #12721), pHistone 3 (Ser10, 1:1,000; #3377), pCHK1 (Ser317, 1:1,000; #12302), KEAP1 (1:1,000; #8047), cleaved Caspase3 (1:1,000; #9661), Caspase3 (1:1,000; #9665), PARP (1:1,000; #9542), CDK6 (1:1,000; # 3136) (Cell Signaling Technology); Cul1 (1:500; #718700), Cul2 (1:250; #7001792), Rbx1 (1:300; #342500) (Invitrogen); Fbxw7 (1:300; # 55290–1-AP), Fbxo22 (1:500; #13606–1-AP) (Proteintech); Cul3 (1:1,000; # A301–109A), CSN5 (1:2,000; #A300–014A) (Bethyl Laboratories); Fbxo3 (1:300; #AP9195) (Abgent); p21 (1:500; #556431) (BD Biosciences); Fbxo30 (1:500; # H00084085-B01) (Abnova); β-Actin (1:5,000; #A5441) (Sigma-Aldrich); Fbxo7 (1:500; #PA527589) (Pierce). Horseradish peroxidase-conjugated secondary antibodies (1:3,000) were purchased from Promega AG and bound antibody was detected by enhanced chemiluminescence (Pierce).

### Cell viability assay

A standardized CellTiter-Glo Assay (Promega #G7570) based on the quantification of ATP was applied according to the manufacturer's protocol to measure the amount of viable cells in culture. In brief, cells were seeded into 384-well plates at optimal density for each cell line. After 4 h, compounds were added at different concentrations in growth medium and incubated for another 72 h in a humidifying chamber at 37 °C and 5% CO_2_. Eventually, equal amounts of CellTiter-Glo reagent were added directly to the cells, incubated for 15 min and luminescence was recorded using PHERAstar plate reader (BMG LABTECH GmbH). The activity of the compounds was calculated relative to vehicle (*A*_max_ 0%) and to 1 μM bortezomib (*A*_max_-100%). Bortezomib induced quantitative cell death in all cell lines used. EC_50_ values were calculated from the plot of percentage of inhibition versus inhibitor concentration using the nonlinear regression analysis software Origin 7.5SR6 (OriginLab Corp.).

### Xenograft experiment

All animal experiments were approved by the Kantonales Veterinäramt Basel-Stadt and were conducted in accordance with the Eidgenössisches Tierschutzgesetz and the Eidgenössische Tierschutzverordnung. SU-DHL-1 cells (1 × 10^7^; DSMZ) were implanted subcutaneously into SCID-bg female 7–9-week-old mice (Taconic) and developing tumours were propagated once before subcutaneous transplantation for efficacy studies. Treatment was initiated when tumours reached 150–200 mm^3^ volume. At this point, animals were randomized into different treatment groups based on similar tumour size and body weight. Randomization was conducted by using an internal computer software (Indigo). CSN5i-3 was formulated in 100 mM citrate buffer pH 3, PEG300, Kolliphor HS15, 1 N HCl (56:30:10:4%w/w). Tumour response is reported as percentage change in tumour volume at the last day of treatment relative to start of treatment. Sample size was *n*=4 (based on IC_50_/EC_50_ data and pharmacokinetic (PK) modelling of the compound) and statistical analysis was performed by comparing the treatment groups (*n*=4) to the vehicle control group (*n*=4) at endpoint by Kruskal–Wallis followed by Dunn's *post hoc* test on ΔTVol. At day 10 of treatment blood, samples were taken and concentrations of CSN5i-3 were determined at indicated time points post dose using ultra-high-pressure liquid chromatography/tandem MS. At the end of treatment, tumours were excised and snap frozen in liquid nitrogen. Frozen tumours were pulverized using CryoPrep (Covaris Inc.) and protein lysates were prepared from tumour powder for immunoblot analysis.

### Data availability

The authors declare that the data supporting the findings of this study are available within the article and its Supplementary Information files. Accession codes: the coordinates for the X-ray structures have been deposited to the Protein Data Bank (PDB) with accession codes 5JOH (complex with CSN5i-1b) and 5JOG (complex with CSN5i-3).

## Additional information

**How to cite this article**: Schlierf, A. *et al*. Targeted inhibition of the COP9 signalosome for treatment of cancer. *Nat. Commun.* **7**, 13166 doi: 10.1038/ncomms13166 (2016).

## Supplementary Material

Supplementary Information Supplementary Figures 1-7 and Supplementary Table 1-2.

Supplementary Data 1 Excel sheets with raw data on Cancer Cell Line Encyclopedia data with CSN5i-2, CSN5i-2e, Bortezomib and MLN4924.

## Figures and Tables

**Table 1:** IC_50_ values [**μ**M] of CSN5 inhibitors against JAMM domain proteases and other metalloproteinases.

Protease	CSN5i-1b	CSN5i-2	CSN5i-2e	CSN5i-3
CSN5*	1.1±0.5 (*n*=14)	0.002±0.001 (*n*=2)	2.1±1.8 (*n*=2)	0.0058±0.0005 (*n*=5)
AMSHLP*	>100	>100	>100	>100
RPN11*	ND	ND	ND	53±26 (*n*=2)
MMP1	ND	ND	ND	>100
MMP2	>100	89±3 (*n*=2)	≥90	>100
MMP8	>100	59±8 (*n*=2)	48±8 (*n*=2)	14.0±0.1 (*n*=2)
MMP9	>100	3.8±0.4 (*n*=2)	3.2±0.2 (*n*=2)	7.4±2.0 (*n*=2)
MMP12	>100	>100	>100	>100
MMP13	>100	>100	>100	>100
MMP14	>100	15.40±0.02 (*n*=2)	11±1 (*n*=2)	4.6±0.5 (*n*=2)
ADAM17	>100	>100	>100	>100

*JAMM domain proteases.

ND, not determined.

Data are means±s.d. of at least two independent experiments. Number of replicates is indicated in brackets.

**Table 2:** CYP3A4 inhibition and key pharmacokinetic parameters in mice of CSN5 inhibitors.

Parameter	CSN5i-2	CSN5i-3
CYP3A4 (IC_50_)	<0.5 μM (*n*=2)	6.8±1.8 μM (*n*=2)
*t*_1/2_*	36±1 min	150±18 min
Cl*	82±25 μl min^−1^ mg^−1^	36±3 μl min^−1^ mg^−1^
F*	<1%	33±2%

Cl, clearance; *F*, absolute bioavailability; *t*_1/2_, half-life.

^*^Pharmacokinetic parameters were determined in mice (*n*=3) following a single intravenous (1 mg per kg body weight) and a single oral (3 mg per kg body weight) dose.

## References

[b1] Hershko A., Ciechanover A. & Varshavsky A. Basic medical research award. The ubiquitin system. Nat. Med. 6, 1073–1081 (2000). 11017125 10.1038/80384

[b2] Coux O., Tanaka K. & Goldberg A. L. Structure and functions of the 20S and 26S proteasomes. Annu. Rev. Biochem. 65, 801–847 (1996). 8811196 10.1146/annurev.bi.65.070196.004101

[b3] Komander D. & Rape M. The ubiquitin code. Annu. Rev. Biochem. 81, 203–229 (2012). 22524316 10.1146/annurev-biochem-060310-170328

[b4] Deshaies R. J. & Joazeiro C. A. RING domain E3 ubiquitin ligases. Annu. Rev. Biochem. 78, 399–434 (2009). 19489725 10.1146/annurev.biochem.78.101807.093809

[b5] Schulman B. A. & Harper J. W. Ubiquitin-like protein activation by E1 enzymes: the apex for downstream signalling pathways. Nat. Rev. Mol. Cell Biol. 10, 319–331 (2009). 19352404 10.1038/nrm2673 PMC2712597

[b6] Ye Y. & Rape M. Building ubiquitin chains: E2 enzymes at work. Nat. Rev. Mol. Cell Biol. 10, 755–764 (2009). 19851334 10.1038/nrm2780 PMC3107738

[b7] Mani A. & Gelmann E. P. The ubiquitin-proteasome pathway and its role in cancer. J. Clin. Oncol. 23, 4776–4789 (2005). 16034054 10.1200/JCO.2005.05.081

[b8] Popovic D., Vucic D. & Dikic I. Ubiquitination in disease pathogenesis and treatment. Nat. Med. 20, 1242–1253 (2014). 25375928 10.1038/nm.3739

[b9] Adams J. & Kauffman M. Development of the proteasome inhibitor Velcade (Bortezomib). Cancer Invest. 22, 304–311 (2004). 15199612 10.1081/cnv-120030218

[b10] Khan M. L. & Stewart A. K. Carfilzomib: a novel second-generation proteasome inhibitor. Future Oncol. 7, 607–612 (2011). 21568676 10.2217/fon.11.42 PMC3931449

[b11] Argyriou A. A., Cavaletti G., Bruna J., Kyritsis A. P. & Kalofonos H. P. Bortezomib-induced peripheral neurotoxicity: an update. Arch. Toxicol. 88, 1669–1679 (2014). 25069804 10.1007/s00204-014-1316-5

[b12] Buac D. . From bortezomib to other inhibitors of the proteasome and beyond. Curr. Pharm. Des. 19, 4025–4038 (2013). 23181572 10.2174/1381612811319220012 PMC3657018

[b13] Nalepa G., Rolfe M. & Harper J. W. Drug discovery in the ubiquitin-proteasome system. Nat. Rev. Drug Discov. 5, 596–613 (2006). 16816840 10.1038/nrd2056

[b14] Skaar J. R., Pagan J. K. & Pagano M. SCF ubiquitin ligase-targeted therapies. Nat. Rev. Drug Discov. 13, 889–903 (2014). 25394868 10.1038/nrd4432 PMC4410837

[b15] Li W . Genome-wide and functional annotation of human E3 ubiquitin ligases identifies MULAN, a mitochondrial E3 that regulates the organelle's dynamics and signaling. PLoS ONE 3, e1487 (2008). 18213395 10.1371/journal.pone.0001487 PMC2198940

[b16] Petroski M. D. & Deshaies R. J. Function and regulation of cullin-RING ubiquitin ligases. Nat. Rev. Mol. Cell Biol. 6, 9–20 (2005). 15688063 10.1038/nrm1547

[b17] Soucy T. A. . An inhibitor of NEDD8-activating enzyme as a new approach to treat cancer. Nature 458, 732–736 (2009). 19360080 10.1038/nature07884

[b18] Zheng N. . Structure of the Cul1-Rbx1-Skp1-F boxSkp2 SCF ubiquitin ligase complex. Nature 416, 703–709 (2002). 11961546 10.1038/416703a

[b19] Jin J. . Systematic analysis and nomenclature of mammalian F-box proteins. Genes Dev. 18, 2573–2580 (2004). 15520277 10.1101/gad.1255304 PMC525538

[b20] Skaar J. R., Pagan J. K. & Pagano M. Mechanisms and function of substrate recruitment by F-box proteins. Nat. Rev. Mol. Cell Biol. 14, 369–381 (2013). 23657496 10.1038/nrm3582 PMC3827686

[b21] Bennett E. J., Rush J., Gygi S. P. & Harper J. W. Dynamics of cullin-RING ubiquitin ligase network revealed by systematic quantitative proteomics. Cell 143, 951–965 (2010). 21145461 10.1016/j.cell.2010.11.017 PMC3008586

[b22] Deshaies R. J., Emberley E. D. & Saha A. Control of cullin-ring ubiquitin ligase activity by nedd8. Subcell Biochem. 54, 41–56 (2010). 21222272 10.1007/978-1-4419-6676-6_4

[b23] Duda D. M. . Structural regulation of cullin-RING ubiquitin ligase complexes. Curr. Opin. Struct. Biol. 21, 257–264 (2011). 21288713 10.1016/j.sbi.2011.01.003 PMC3151539

[b24] Pierce N. W. . Cand1 promotes assembly of new SCF complexes through dynamic exchange of F box proteins. Cell 153, 206–215 (2013). 23453757 10.1016/j.cell.2013.02.024 PMC3656483

[b25] Walden H., Podgorski M. S. & Schulman B. A. Insights into the ubiquitin transfer cascade from the structure of the activating enzyme for NEDD8. Nature 422, 330–334 (2003). 12646924 10.1038/nature01456

[b26] Cope G. A. & Deshaies R. J. COP9 signalosome: a multifunctional regulator of SCF and other cullin-based ubiquitin ligases. Cell 114, 663–671 (2003). 14505567 10.1016/s0092-8674(03)00722-0

[b27] Wei N. & Deng X. W. The COP9 signalosome. Annu. Rev. Cell Dev. Biol. 19, 261–286 (2003). 14570571 10.1146/annurev.cellbio.19.111301.112449

[b28] Wolf D. A., Zhou C. & Wee S. The COP9 signalosome: an assembly and maintenance platform for cullin ubiquitin ligases? Nat. Cell Biol. 5, 1029–1033 (2003). 14647295 10.1038/ncb1203-1029

[b29] Ambroggio X. I., Rees D. C. & Deshaies R. J. JAMM: a metalloprotease-like zinc site in the proteasome and signalosome. PLoS Biol. 2, E2 (2004). 14737182 10.1371/journal.pbio.0020002 PMC300881

[b30] Lingaraju G. M. . Crystal structure of the human COP9 signalosome. Nature 512, 161–165 (2014). 25043011 10.1038/nature13566

[b31] Cope G. A. & Deshaies R. J. Targeted silencing of Jab1/Csn5 in human cells downregulates SCF activity through reduction of F-box protein levels. BMC Biochem. 7, 1 (2006). 16401342 10.1186/1471-2091-7-1 PMC1360668

[b32] Lee Y. H. . Molecular targeting of CSN5 in human hepatocellular carcinoma: a mechanism of therapeutic response. Oncogene 30, 4175–4184 (2011). 21499307 10.1038/onc.2011.126 PMC3140552

[b33] Pan Y. & Claret F. X. Targeting Jab1/CSN5 in nasopharyngeal carcinoma. Cancer Lett. 326, 155–160 (2012). 22867945 10.1016/j.canlet.2012.07.033 PMC3474602

[b34] Zhong G., Li H., Shan T. & Zhang N. CSN5 silencing inhibits invasion and arrests cell cycle progression in human colorectal cancer SW480 and LS174T cells *in vitro*. Int. J. Clin. Exp. Pathol. 8, 2809–2815 (2015). 26045788 PMC4440097

[b35] Lee M. H., Zhao R., Phan L. & Yeung S. C. Roles of COP9 signalosome in cancer. Cell Cycle 10, 3057–3066 (2011). 21876386 10.4161/cc.10.18.17320 PMC3218617

[b36] Richardson K. S. & Zundel W. The emerging role of the COP9 signalosome in cancer. Mol. Cancer Res. 3, 645–653 (2005). 16380502 10.1158/1541-7786.MCR-05-0233 PMC2943958

[b37] Shackleford T. J. & Claret F. X. JAB1/CSN5: a new player in cell cycle control and cancer. Cell Div. 5, 26 (2010). 20955608 10.1186/1747-1028-5-26 PMC2976740

[b38] Cope G. A. . Role of predicted metalloprotease motif of Jab1/Csn5 in cleavage of Nedd8 from Cul1. Science 298, 608–611 (2002). 12183637 10.1126/science.1075901

[b39] Hassiepen U. . A sensitive fluorescence intensity assay for deubiquitinating proteases using ubiquitin-rhodamine110-glycine as substrate. Anal. Biochem. 371, 201–207 (2007). 17869210 10.1016/j.ab.2007.07.034

[b40] Fischer E. S. . The molecular basis of CRL4DDB2/CSA ubiquitin ligase architecture, targeting, and activation. Cell 147, 1024–1039 (2011). 22118460 10.1016/j.cell.2011.10.035

[b41] Kikuchi K., Ishii N., Asao H. & Sugamura K. Identification of AMSH-LP containing a Jab1/MPN domain metalloenzyme motif. Biochem. Biophys. Res. Commun. 306, 637–643 (2003). 12810066 10.1016/s0006-291x(03)01009-x

[b42] Verma R. . Role of Rpn11 metalloprotease in deubiquitination and degradation by the 26S proteasome. Science 298, 611–615 (2002). 12183636 10.1126/science.1075898

[b43] Oladghaffari M., Islamian J. P., Baradaran B. & Monfared A. S. MLN4924 therapy as a novel approach in cancer treatment modalities. J. Chemother. 28, 74–82 (2015). 10.1179/1973947815Y.0000000066 26292710

[b44] Galan J. M. & Peter M. Ubiquitin-dependent degradation of multiple F-box proteins by an autocatalytic mechanism. Proc. Natl Acad. Sci. USA 96, 9124–9129 (1999). 10430906 10.1073/pnas.96.16.9124 PMC17743

[b45] Wirbelauer C. . The F-box protein Skp2 is a ubiquitylation target of a Cul1-based core ubiquitin ligase complex: evidence for a role of Cul1 in the suppression of Skp2 expression in quiescent fibroblasts. EMBO J. 19, 5362–5375 (2000). 11032804 10.1093/emboj/19.20.5362 PMC314004

[b46] Zhou P. & Howley P. M. Ubiquitination and degradation of the substrate recognition subunits of SCF ubiquitin-protein ligases. Mol. Cell 2, 571–580 (1998). 9844630 10.1016/s1097-2765(00)80156-2

[b47] Barretina J. . The Cancer Cell Line Encyclopedia enables predictive modelling of anticancer drug sensitivity. Nature 483, 603–607 (2012). 22460905 10.1038/nature11003 PMC3320027

[b48] Meziane E., Randle S. J., Nelson D. E., Lomonosov M. & Laman H. Knockdown of Fbxo7 reveals its regulatory role in proliferation and differentiation of haematopoietic precursor cells. J. Cell Sci. 124, 2175–2186 (2011). 21652635 10.1242/jcs.080465

[b49] Nelson D. E., Randle S. J. & Laman H. Beyond ubiquitination: the atypical functions of Fbxo7 and other F-box proteins. Open Biol. 3, 130131 (2013). 24107298 10.1098/rsob.130131 PMC3814724

[b50] Epstein A. L. & Kaplan H. S. Biology of the human malignant lymphomas. I. Establishment in continuous cell culture and heterotransplantation of diffuse histiocytic lymphomas. Cancer 34, 1851–1872 (1974). 4140017 10.1002/1097-0142(197412)34:6<1851::aid-cncr2820340602>3.0.co;2-4

[b51] Nagel S. . Amplification at 7q22 targets cyclin-dependent kinase 6 in T-cell lymphoma. Leukemia 22, 387–392 (2008). 17989712 10.1038/sj.leu.2405028

[b52] Moreau P. . Proteasome inhibitors in multiple myeloma: 10 years later. Blood 120, 947–959 (2012). 22645181 10.1182/blood-2012-04-403733 PMC4123429

[b53] Robak T. Bortezomib in the treatment of mantle cell lymphoma. Future Oncol. 11, 2807–2818 (2015). 26347482 10.2217/fon.15.191

[b54] Enchev R. I., Schulman B. A. & Peter M. Protein neddylation: beyond cullin-RING ligases. Nat. Rev. Mol. Cell Biol. 16, 30–44 (2015). 25531226 10.1038/nrm3919 PMC5131867

[b55] Cooper E. M. . K63-specific deubiquitination by two JAMM/MPN+ complexes: BRISC-associated Brcc36 and proteasomal Poh1. EMBO J. 28, 621–631 (2009). 19214193 10.1038/emboj.2009.27 PMC2666030

[b56] Zheng H. . A BRISC-SHMT complex deubiquitinates IFNAR1 and regulates interferon responses. Cell Rep. 5, 180–193 (2013). 24075985 10.1016/j.celrep.2013.08.025 PMC3813903

[b57] Py B. F., Kim M. S., Vakifahmetoglu-Norberg H. & Yuan J. Deubiquitination of NLRP3 by BRCC3 critically regulates inflammasome activity. Mol. Cell 49, 331–338 (2013). 23246432 10.1016/j.molcel.2012.11.009

[b58] Kroemer M., Dreyer M. K. & Wendt K. U. APRV - a program for automated data processing, refinement and visualization. Acta. Crystallogr. D Biol. Crystallogr. 60, 1679–1682 (2004). 15333953 10.1107/S0907444904015215

[b59] McCoy A. J. . Phaser crystallographic software. J. Appl. Crystallogr. 40, 658–674 (2007). 19461840 10.1107/S0021889807021206 PMC2483472

[b60] Echalier A. . Insights into the regulation of the human COP9 signalosome catalytic subunit, CSN5/Jab1. Proc. Natl Acad. Sci. USA 110, 1273–1278 (2013). 23288897 10.1073/pnas.1209345110 PMC3557056

[b61] Emsley P. & Cowtan K. Coot: model-building tools for molecular graphics. Acta Crystallogr. D Biol. Crystallogr. 60, 2126–2132 (2004). 15572765 10.1107/S0907444904019158

