# Supplementary material for: Targeted inhibition of the COP9 signalosome for treatment of cancer
Source: Nat Commun. 2016 Oct 24;7:13166. doi: 10.1038/ncomms13166 (PMC5078989; doi:10.1038/ncomms13166)
Supplement: Supplementary Information — Supplementary Figures 1-7 and Supplementary Table 1-2. [file ncomms13166-s1.pdf]

Supplementary Figure 1

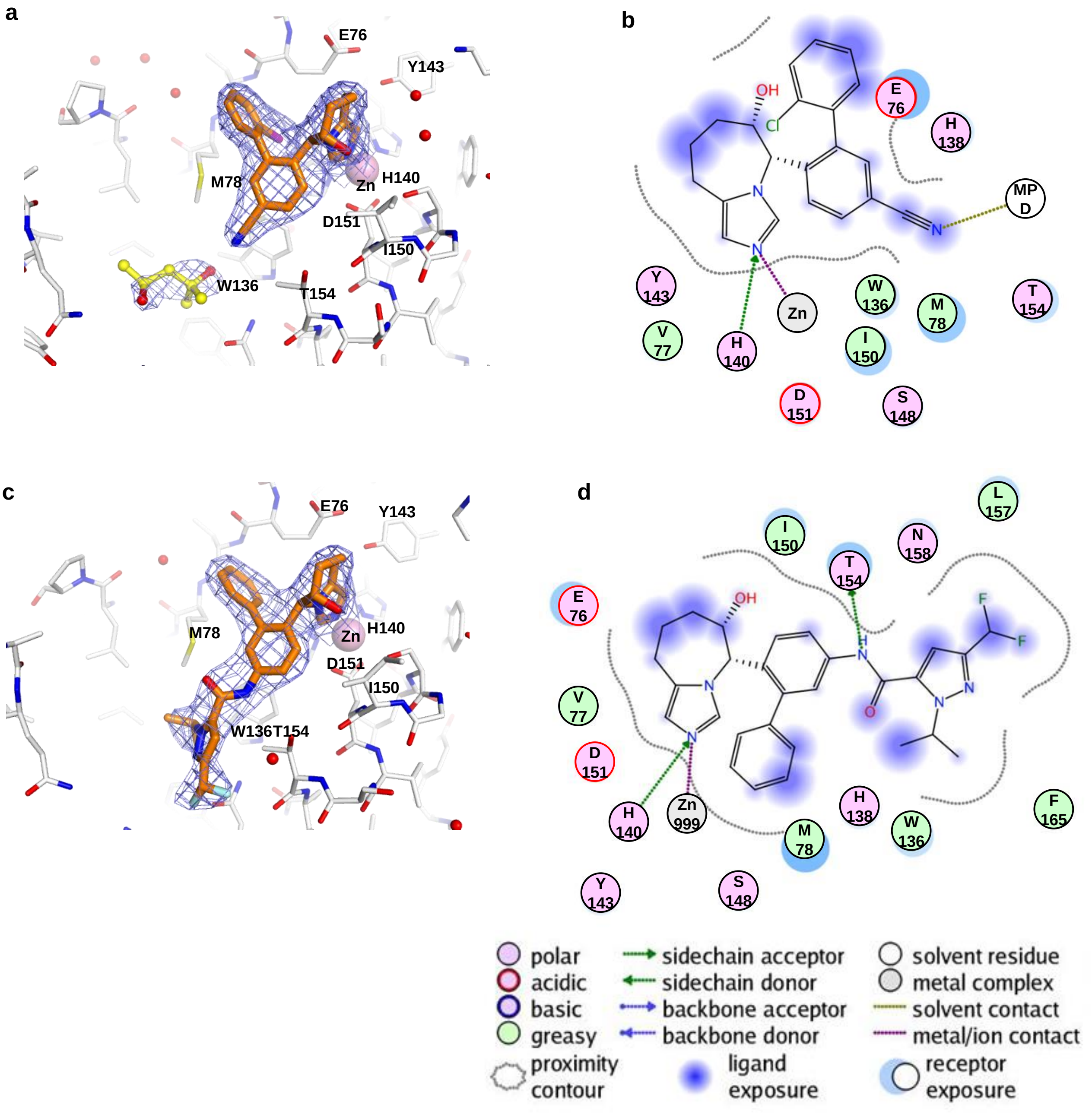

Supplementary Figure 1 | CSN5i ligands bind to the catalytic site of CSN5. (a) 2Fo-Fc electron density of CSN5i-1b and the putative methylpentandiol buffer molecule in yellow. **(b)** Ligand interaction diagram for CSN5i-1b. **(c)** 2Fo-Fc electron density of CSN5i-3. **(d)** Ligand interaction diagram for CSN5i-3. The electron density figures were generated using PyMOL and the ligand interaction plots using the algorithm described by Clark et al. (Clark, A. M., Labute, P. & Santavy, M. 2D Structure Depiction. *J. Chem. Inf. Model.* **46**, 1107-1123 (2006)).

Supplementary Figure 2

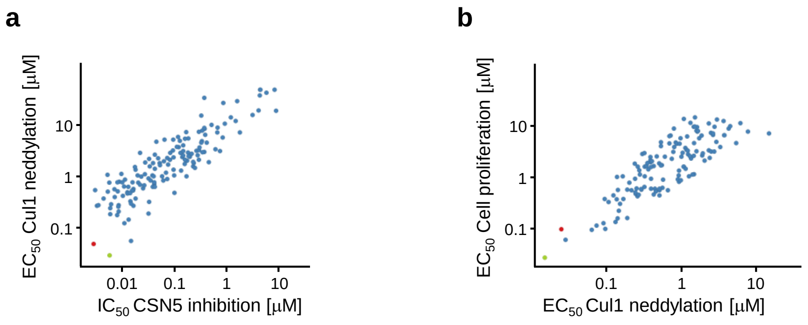

Supplementary Figure 2 | CSN5 inhibition correlates with accumulation of neddylated Cul1 and cell proliferation for different derivatives of the tetrahydro-imidazoazepinol chemotype. (a) IC_{50} values for CSN5 inhibition versus EC_{50} values for accumulation of neddylated Cul1 in MIA PaCa-2 cells. CSN5i-2 is marked with a red, CSN5i-3 with a green dot. Data are means of n $\geq$ 2 independent experiments. MIA PaCa-2 cells (ATCC #CRL-1420) were cultured in DMEM medium (GIBCO #61965-059) supplemented with 10% FBS. Neddylated Cul1 was detected using Meso Scale Discovery® electrochemiluminescence detection technology. For this, cells were pretreated for 3 h with 400 nM MLN4924 and, after a washing step, CSN5 inhibitor was added and cells were incubated for another 3 h. Finally, cells were prepared for MSD-based capture-ELISA according to standard protocol with capture mouse anti-Cul1 antibody (Invitrogen #32-2400) and detection rabbit anti-Nedd8 antibody (Epitomics #1571-1). Note that the systematic shift between biochemical and cellular IC_{50}/EC_{50} values may result from different assay conditions (e.g. the presence of serum in the cell culture coupled with unspecific plasma protein binding of compounds) or from inherent assay differences (e.g. different substrate concentrations). (**b**) EC_{50} values for inhibition of HCT116 cell proliferation versus EC_{50} values for accumulation of neddylated Cul1. CSN5i-2 is marked with a red, CSN5i-3 with a green dot.

Supplementary Figure 3

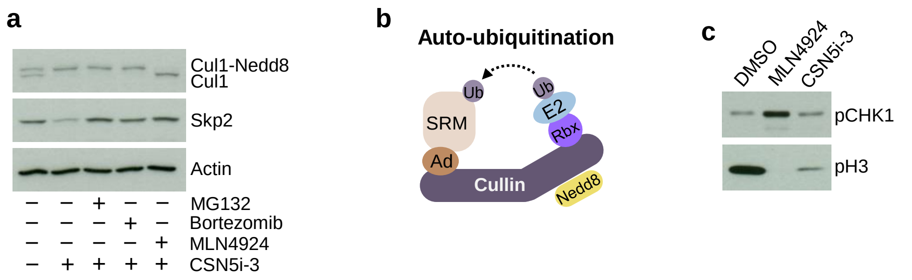

Supplementary Figure 3 | Trapping SCF^{Skp2} in the neddylated state facilitates degradation of Skp2 by the proteasome. (**a**) Immunoblot for Cul1 and Skp2 after pre-treatment of HCT116 cells for 30 min with 2 μM MG132, 0.5 μM bortezomib or 1 μM MLN4924 as indicated, followed by treatment with 1 μM CSN5i-3 for 4 h. **(b)** Schematic illustration of CRL auto-ubiqiuitination when substrates are exhausted and CRLs trapped in the neddylated conformation (see also Fig. 1a). **(c)** Immunoblot for pCHK1 and pH3 after treatment of HCT116 cells with 1 μM MLN4924 or 1 μM CSN5i-3.

Supplementary Figure 4

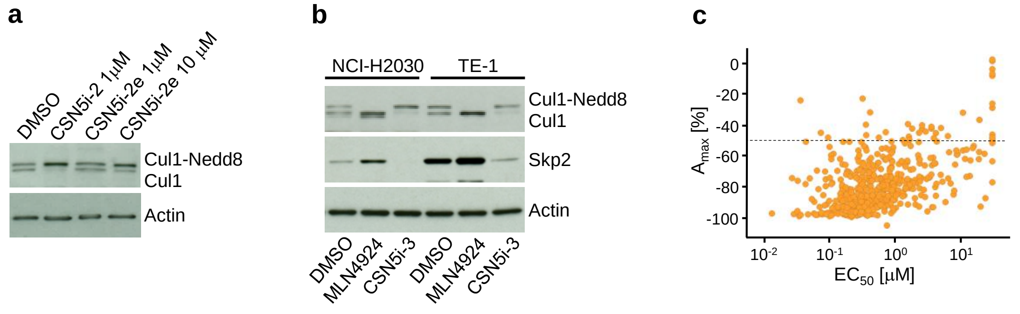

Supplementary Figure 4 | Mechanistic effect of CSN5 inhibitors. (**a**) Immunoblot for Cul1 after treatment of HCT116 cells with CSN5i-2 and the *R,R*-enantiomer CSN5i-2e at the indicated concentrations. **(b)** Immunoblot for Cul1 and Skp2 after treatment of NCI-H2030 and TE-1 cells with 1 µM MLN4924 or 1 µM CSN5i-3. **(c)** Concentration dependent effects of MLN4924 on a panel of cancer cell lines. Shown are the high-concentration effect level (A_{max}) as derived from the sigmoidal fit to the individual dose-response curves and the corresponding transitional concentration values (EC_{50}).

Supplementary Figure 5

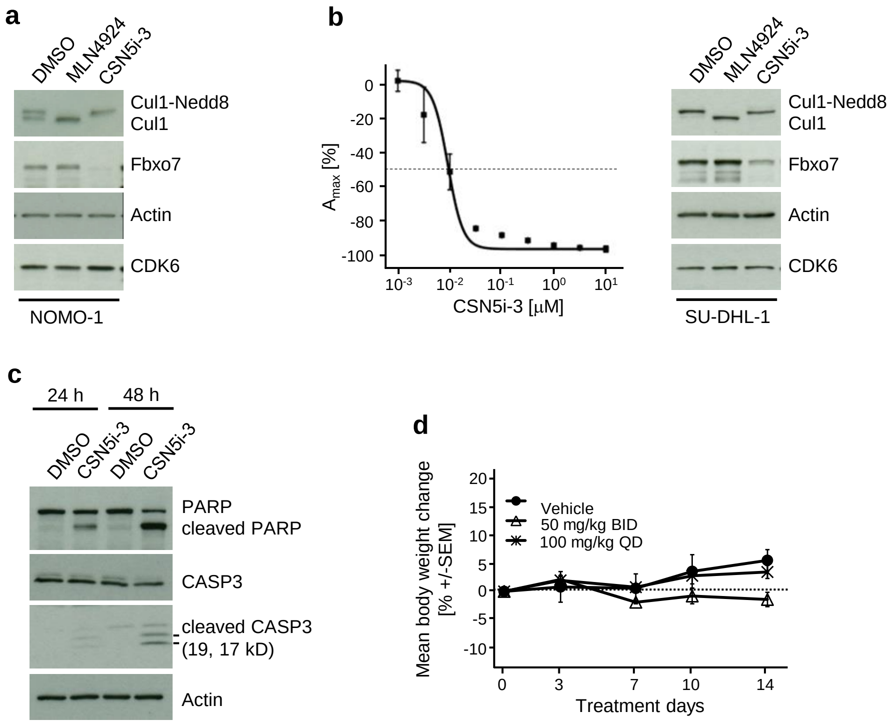

Supplementary Figure 5 | Additional data on the xenograft study with SU-DHL-1 cells. (**a**) CSN5 inhibition results in donwregulation of FBXO7 in NOMO-1 cells. Imunoblot for Cul1, Fbxo7 and CDK6 after treatment with 1 μM MLN4924 or 1 μM CSN5i-3. **(b)** Drug response for CSN5i-3 on SU-DHL-1 cells (n=2, ± SD), and immunoblot for Cul1, Fbxo7 and CDK6 after treatment with 1 μM MLN4924 or 1 μM CSN5i-3. **(c)** Treatment of SU-DHL-1 cell lines with 1 μM CSN5i-3 for 24 h or 48 h and immunoblot for PARP, caspase 3 and cleaved caspase 3. (**d**) Mean body weight changes of mice from xenograft study are shown ± SEM (n=4).

Supplementary Figure 6

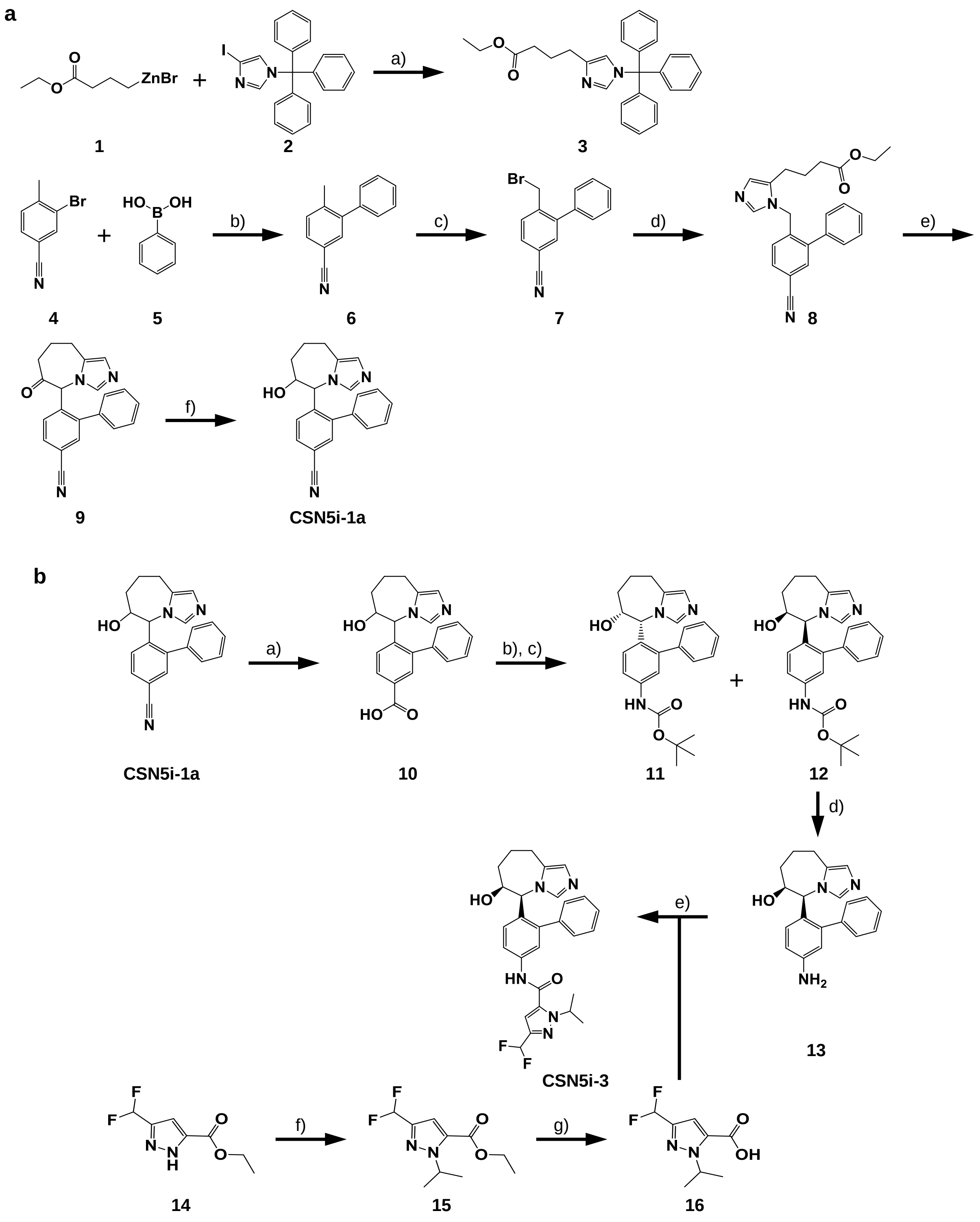

Supplementary Figure 6 | Synthesis of CSN5 inhibitors. (**a**) Synthesis of CSN5i-1a. a) $PdCl_2(dppf){\cdot}CH_2Cl_2$, THF, 0 - 70°C, 73%. b) $PdCl_2(dppf).CH_2Cl_2$, Cs_2CO_3, DMF/H_2O (1:1), 90°C, 97%. c) NBS, AIBN, CCl_4, 80°C, 68%. d) i) **3**, CH_3CN, rt, ii) MeOH, 70°C, 94%. e) KO*t*Bu, THF, rt, 84%. f) $NaBH_4$, MeOH, 0°C, 94%. . (**b**) Synthesis of CSN5i-3. a) NaOH, THF/MeOH (1:1), 60°C, 78%. b) i) DPPA, TEA, THF, rt, ii) *t*-butanol, 80°C, 68%. c) Chiral separation by preparative chiral HPLC chromatography on a Chiralpak IC column. d) 4M HCl/dioxane, rt, 91%. e) T3P, DIPEA, DCM, rt, 74%. f) 2-iodopropane, K_2CO_3, DMF, 40°C, 75%. g) $LiOH{\cdot}H_2O$, THF/MeOH/H_2O (1:1:1), rt, 99%.

Supplementary Figure 7

Fig. 1e

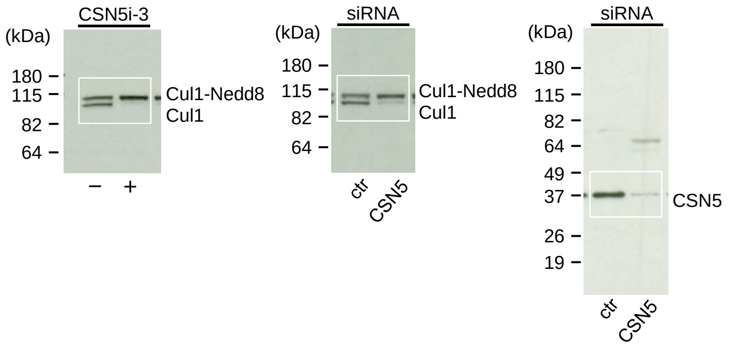

Fig. 2a

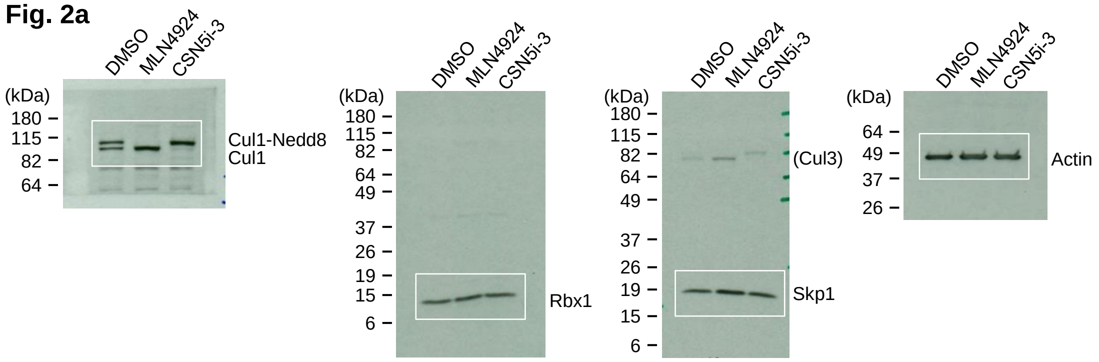

Fig. 2b

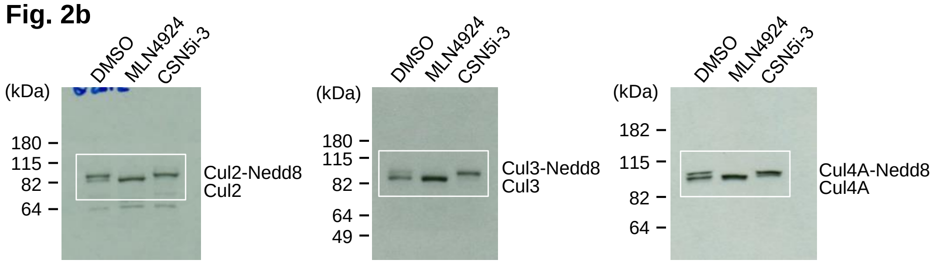

Fig. 2c

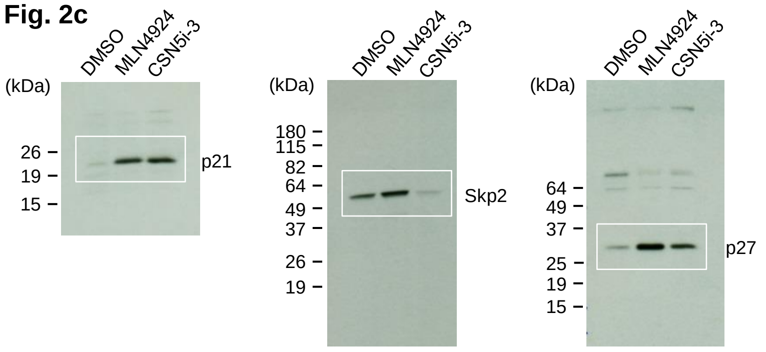

Fig. 2d

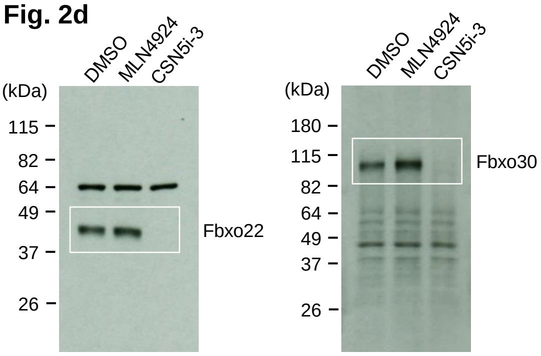

Fig. 2e

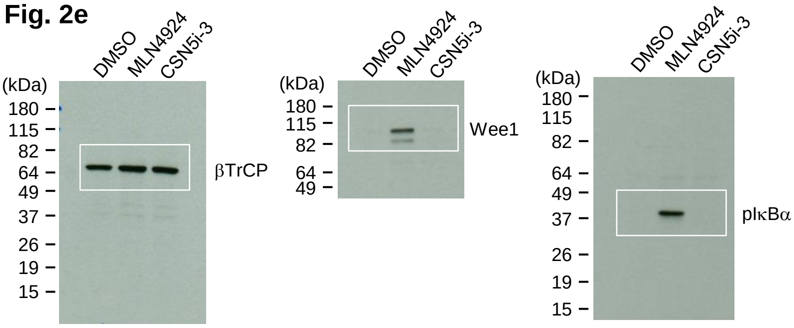

Fig. 2f

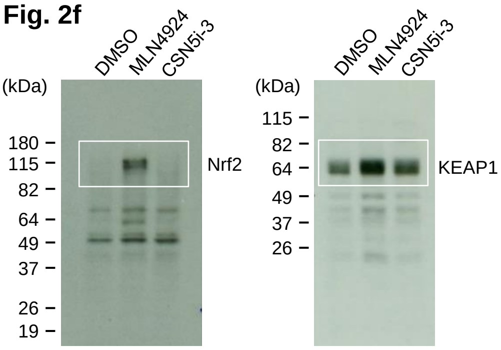

Fig. 2g

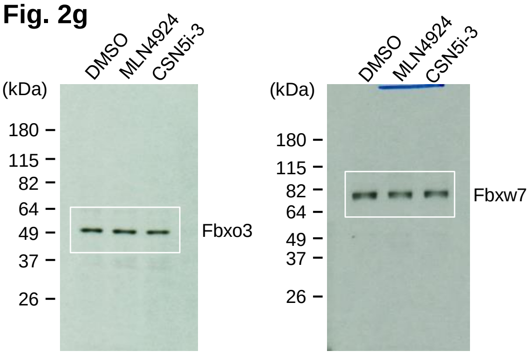

Fig. 4b

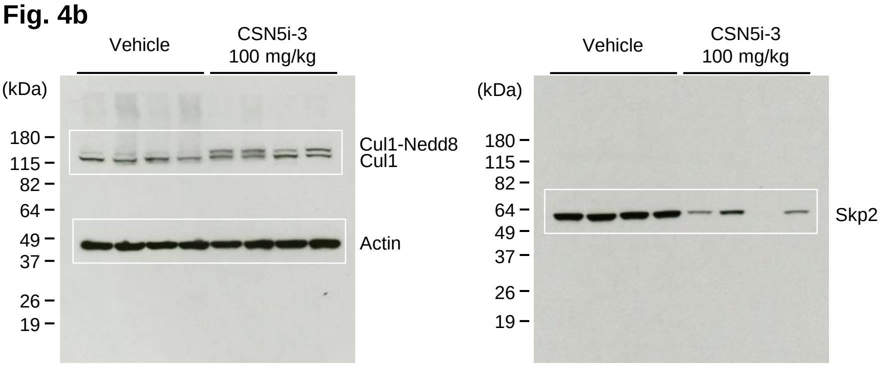

Supplementary Figure 7 | Full-scanned images of western blots shown in the main figures.

Supplementary Table 1

**Supplementary Table 1 | Small-molecule library and screening data.**		
**Category**	**Parameter**	**Description**
Assay	Nature of the assay	TR-FRET-based protease activity assay.
	Assay strategy	Identify COPS9 signalosome inhibitors.
	Reagents and sources	DMSO (Cat. No. 41644), Tris (Cat. No. T2663), TCEP (Cat. No. C4706), NaCl (Cat. No.71976) and BSA (Cat. No. A3059) by Sigma-Aldrich Chemie GmbH, Buchs, Switzerland; Pluronic F127 (Cat. No. P6866), terbium-labelled anti-His antibody (Cat-No PV3544) was provided by Invitrogen, Paisley, UK; 1536-well format white polystyrene plates (Cat. No.789175-A) by Greiner via Huberlab AG, Aesch, Switzerland; Signalosome complex (batch No. EPP-110126-cl-COP9 (SBM)), Alexa488-neddylated cullin-4 complex (batch No. 181) were prepared in-house.
	Assay protocol	High throughput assay determining the deneddylating activity of the proteolytic signalosome subunit COPS5 by measuring the TR-FRET signal from a His-tagged cullin/Rbx complex that had been conjugated to Nedd8 labelled with Alexa 488.
Library	Library size	~1,500,000
	Source	Novartis compound library
	Additional comments	Compound stocks at 2 mM in 90% DMSO
Screen	Format	1536-well
	Concentration(s) tested	25 μM
	Plate controls	Assay buffer; DMSO
	Reagent/ compound dispensing system	Compound transfer into 1536-well plates with Hummingbird system (obtained from Genomic Solutions Ltd., Huntingdon, Cambridgeshire, U. K. through Zinsser Analytic, Frankfurt, Germany); MarkIII automated screening system (Evotec Technologies, Hamburg, now part of PerkinElmer).
	Detection instrument and software	EnVision (Perkin Elmer)
	Assay validation/QC	Average Z’ values for the primary screen: 0.8 ± 0.1
Post-HTS analysis	Hit criteria	Inhibition of $\geq$30%
	Hit rate	Overall 1.02 %
	Structure confirmation	Compound structure verified by analytical chemistry methods.
	Hit validation	Dose-response curves in biochemical CSN5 assay and cul1 neddylation in HCT116 cells; x-ray co-crystal structure.

Supplementary Table 2

**Supplementary Table 2 | Crystallographic data of CSN5 crystals in complex with CSN5i-1b or CSN5i-3.**		
**Compound**	**CSN5i-1b**	**CSN5i-3**
PDB	5joh	5jog
**Diffraction Data**		
Wavelength (Å)	1.0	1.0
Space group	I4	I4
a, b, c (Å)	102.1, 102.1, 68,7	101.4, 101.4, 68.6
α, β, γ (°)	90, 90, 90	90, 90, 90
Resolution range (Å)	72-1.99 (1.988-1.994)	57-2.46 (2.47-2.46)
Unique reflections	24374 (260)	12742 (146)
R_{merge} (%)	4.1 (83.2)	4.6 (57.0)
Mean I/sigma (I)	22.5 (2.7)	26.5 (2.5)
Completeness (%)	99.9 (100)	99.9 (100)
Redundancy	6.9 (7.1)	6.8 (6.6)
**Refinement**		
Resolution range (Å)	57.01-1.99 (2.08-1.99)	56.81-2.46 (2.69-2.46)
R_{work} (%)	22.6 (28.2)	20.8 (23.2)
R_{free} (%)	24.8 (29.7)	24.3 (29.3)
Number of non-hydrogen atoms		
Macromolecule	1762	1762
Zn ion	1	1
Water	29	27
Ligand	26	37
R.m.s deviation		
Bond length (Å)	0.01	0.01
Bond angles (°)	0.83	1.16
Ramachandran		
Favored (%)	90.0	90.0
Allowed (%)	8.9	8.9
Generously (%)	1.1	1.1
